# Social intolerance is a consequence, not a cause, of dispersal in spiders

**DOI:** 10.1371/journal.pbio.3000319

**Published:** 2019-07-02

**Authors:** Violette Chiara, Felipe Ramon Portugal, Raphael Jeanson

**Affiliations:** 1 Centre de Recherches sur la Cognition Animale, Centre de Biologie Intégrative, Université de Toulouse, CNRS, UPS, Toulouse, France; 2 Innovations thérapeutiques et résistances, Ecole Nationale Vétérinaire de Toulouse, INRA, Toulouse, France; Macquarie university, AUSTRALIA

## Abstract

From invertebrates to vertebrates, a wealth of species display transient sociality during their life cycle. Investigating the causes of dispersal in temporary associations is important to better understand population dynamics. It is also essential to identify possible mechanisms involved in the evolutionary transition from transient to stable sociality, which has been documented repeatedly across taxa and typically requires the suppression of dispersal. In many animals, the onset of dispersal during ontogeny coincides with a sharp decline in social tolerance, but the causal relationship still remains poorly understood. Spiders offer relevant models to explore this question, because the adults of the vast majority of species (>48,000) are solitary and aggressive, but juveniles of most (if not all) species are gregarious and display amicable behaviors. We deployed a combination of behavioral, chemical, and modelling approaches in spiderlings of a solitary species to investigate the mechanisms controlling the developmental switch leading to the decline of social cohesion and the loss of tolerance. We show that maturation causes an increase in mobility that is sufficient to elicit dispersal without requiring any change in social behaviors. Our results further demonstrate that social isolation following dispersal triggers aggressiveness in altering the processing of conspecifics’ cues. We thus provide strong evidence that aggression is a consequence, not a cause, of dispersal in spiderlings. Overall, this study highlights the need of extended social interactions to preserve tolerance, which opens new perspectives for understanding the routes to permanent sociality.

## Abstract 2

Behavioral experiments with the spider Agelena labyrinthica, coupled to computational modelling, show that an increase in mobility with age drives dispersal in gregarious spiderlings of a solitary species and that the subsequent social isolation triggers aggression.

## Introduction

Natal dispersal is a fundamental life-history trait with profound consequences on both individual fitness and population dynamics [1]. In many taxa of invertebrates and vertebrates, the females lay a clutch of eggs, and after hatching, the offspring display a transient phase of aggregation [2–8]. Group living at the earliest developmental stages can grant juveniles benefits via a reduction of predation risk, more efficient defense, improved development, and facilitated feeding [9–16]. The shift from group to solitary living during ontogeny implies that the advantages of gregariousness are outweighed by the costs, which can result, for instance, from increased competition as individuals mature and grow [15,17–19]. In transient group-living species, natal dispersal is therefore concomitant with the dissolution of the aggregate.

Deciphering how and why juveniles disperse is of critical importance for understanding the transition to more advanced forms of sociality in arthropods. Indeed, one primary route to permanent sociality in insects and arachnids involves the suppression of dispersal in juveniles (e.g., subsocial route) [20–22]. However, the proximal mechanisms driving the decline of social cohesion and the onset of dispersal in temporary group-living arthropods are still poorly understood.

Spiders offer a relevant model to explore this question. The vast majority of the 48,000 species described so far are solitary at adulthood, but transient gregariousness at the earliest developmental stages is a common, if not universal, life-history trait in spiders [4,23,24] (S1 Table). The duration of the gregarious phase in spiderlings is a plastic trait, which depends on the interplay between processes associated with maturation and the response to environmental fluctuations, notably in food availability [25–27]. Interestingly, about 30 species of spiders have developed a permanent social life characterized by the existence of cooperative brood care, hunting, and web building [28]. Eighteen independent derivations of sociality have been identified in spiders [21], suggesting the existence of conserved and robust mechanisms at the origins of sociality in this taxa. A favored route to permanent sociality in spider implies a loss of premating dispersal and involves the extension of social cohesion among spiderlings [21,28]. It is therefore important to unravel what mechanisms trigger the loss of social cohesion in species lacking permanent sociality. Most earlier work on dispersal behaviors in solitary spiderlings investigated the influence of habitat quality on dispersal strategies [29]. It was shown, for instance, that the decision of spiderlings of *Erigone atra* to engage in short-distance (rappelling) or long-distance (ballooning) dispersal depended on the temperature experienced during early development [30]. Very little is known, however, on how the entire groups of spiderlings may disband in the first place. One likely reason for this is that the gregarious stage in solitary spiderlings is so transient that it attracted very little research attention [31].

The transition to permanent sociality not only requires the suppression of dispersal but also the maintenance of tolerance. Indeed, spiderlings at their earliest developmental stage show mutual attraction and social tolerance. This strongly contrasts with postdispersal spiders of solitary species that behave aggressively towards conspecifics and where agonistic interactions can escalate to cannibalism [32]. The concomitant decline in social cohesion and the loss of tolerance in solitary spiders invites questions regarding the causal relationship. Because dispersal is accompanied by social intolerance, it is sometimes believed that the progressive changes in the nature of social interactions trigger group dissolution [33,34]. However, these conclusions often rely on circumstantial evidence based on coincidental observations, and empirical evidence for a causal relationship between dispersal and aggression is limited and mixed [22].

Here, we investigated the proximal mechanisms of dispersal in gregarious spiderlings of the European solitary web-building spider *Agelena labyrinthica*. The genus *Agelena* comprises 60 species, including two social species: *A*. *consociata* and *A*. *republicana* [28]. Like approximately half of spider species in temperate latitudes [35], females of *A*. *labyrinthica* lay a cocoon in late summer and juveniles emerge the next spring after a winter diapause of several months.

In the present study, our first aim was to identify the proximal mechanisms driving the decline in social cohesion. We combined experimental and modelling approaches to elucidate whether the initiation of dispersal requires changes in the nature of interactions among siblings or whether, in contrast, an alternative mechanism, independent of the social context, can explain group dissolution. We next deployed a series of behavioral assays along with the characterization of the chemical signatures of individuals to ask what conditions are required for the onset of aggressiveness in spiderlings. We tested the hypothesis that the sharp decline in social tolerance observed in spiderlings resulted from social isolation consecutive to dispersal. Our overarching goal was to determine whether the loss of social tolerance is a cause or a consequence of dispersal in spiderlings.

## Results

### Onset of dispersal

**Fig 1:**
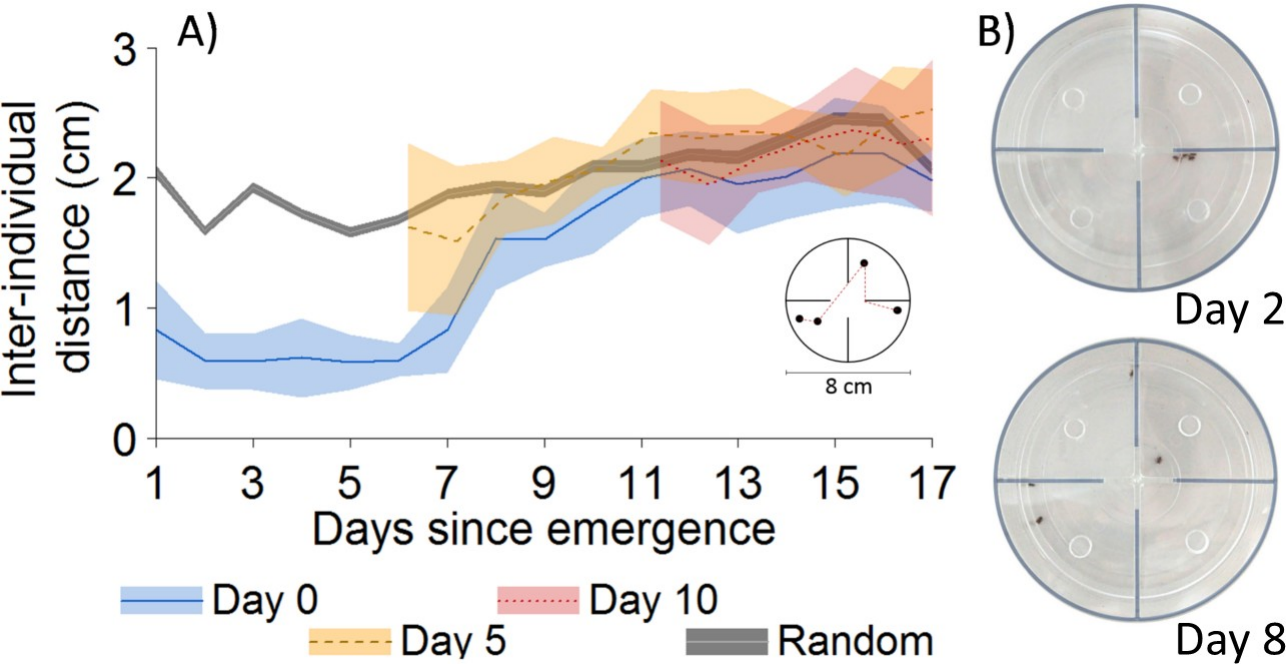
Dynamics of dispersal in spiderlings of *A*. *labyrinthica*. A) Mean interindividual distance as a function of days in groups of four spiderlings introduced to an experimental arena immediately upon emergence (blue, *N* = 10) or five days (yellow, *N* = 10) and 10 days (red, *N* = 9) after emergence. The grey line represents the interindividual distance expected under a random distribution of four individuals. Colored bands are 95% CI. Inset: Spiderlings were introduced in a circular arena partitioned into four compartments. For groups, we computed the interindividual distance as the shortest distance to connect all four individuals (red dotted line). B) Photos representing the spatial distribution of spiderlings in the experimental arena before (Day 2) and after dispersal (Day 8). The inner and outer walls of the arena are highlighted in dark blue on the photos for illustrative purposes. Relevant data values are included in S1 Data.

We first aimed to monitor the onset of dispersal in gregarious spiderlings. We introduced groups of four spiderlings in 4-compartment experimental arenas for 17 consecutive days. Spiderlings initially formed a stable and dense cluster for the first five days (Fig 1A and 1B). From Day 5 onwards, the interindividual distance progressively increased until a value corresponding to the distance expected if individuals were randomly distributed. We needed to verify that the initial aggregation was not a response to a novel and stressful environment and that the increase in interindividual distances did not result from an acclimation to the set-up. To this end, we maintained spiderlings in breeding dishes for five or 10 days before their introduction to experimental arenas and we found that they displayed no initial phase of aggregation (Fig 1A). Also, the periodic removal of silk had no impact on the dynamics of dispersal (S1 Fig). This confirmed that the progressive decline in social cohesion resulted from maturational processes that occurred in the first five to six days following emergence.

### Spiderlings exhibit a developmental switch in locomotion

**Fig 2:**
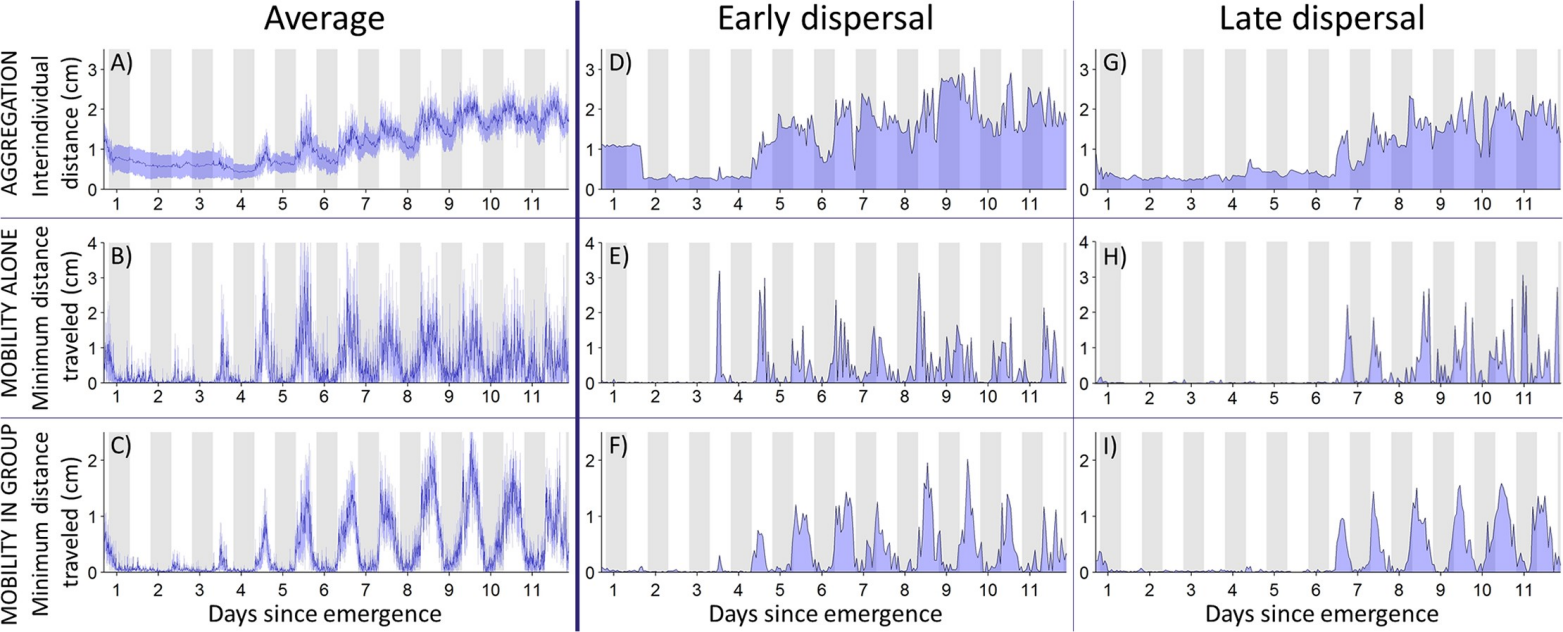
Within-cocoon synchronization and between cocoons variability in the onset of locomotion. The interindividual distance and the level of mobility were determined from the spatial coordinates of spiderlings introduced to dispersal arenas photographed every 10 min for 12 consecutive days. (A) Average (± 95% CI, *N* = 14) mean interindividual distances in groups of four spiderlings introduced to dispersal arenas. (B-C) Average (± 95% CI) activity level of siblings tested alone (B) (*N* = 14) or in groups (C) (*N* = 14). (D to I) Interindividual distance and mobility level in spiderlings from two representative cocoons differing in their timing of dispersal. Relevant data values are included in S2 Data. The grey shading indicates nocturnal phases.

We next examined how ontogeny influences mobility, as the onset of dispersal is accompanied by an increase in locomotion. Individuals introduced alone or in groups to experimental arenas remained mostly motionless for about five days before they gradually increased their mobility over time (Fig 2). In addition, a significant correlation in the first burst of activity was found between siblings tested alone or in groups (Figs 2 and S2; Spearman's rank correlation: *S* = 933, *rho* = 0.47, *P* = 0.026). This showed that social interactions played no role in the increase in mobility observed in dispersal arenas.

### Mutual attraction does not decline over time

**Fig 3:**
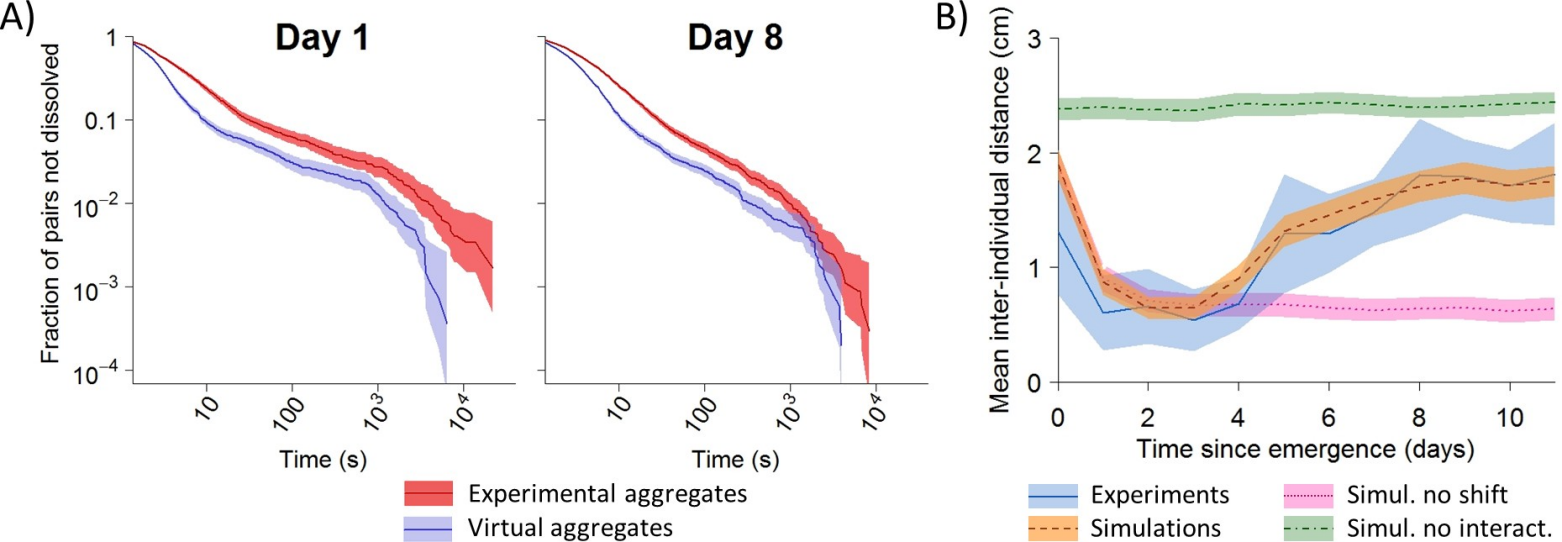
Dispersal results from an increase in mobility and not from a change in mutual attraction. A) Survival curves of the lifetime of aggregates in experimental (red) and virtual (blue) pairs of spiderlings before (Day 1) or after dispersal (Day 8). No difference in hazard ratios (i.e., ratios between extinction rates of experimental and theoretical distributions). [95% CI] was demonstrated over time (Day 1 = 1.52 [1.43–1.62], Day 8 = 1.47 [1.41–1.53]). Colored bands represent 95% CI. Relevant data values are included in S1 Data. B) Dynamics of dispersal in experiments (blue, *N* = 14) and simulations (*N* = 1,000 for each condition) implementing no developmental shift in mobility (pink), no interattraction (green) or, in contrast, incorporating these two rules (orange). Colored bands are 95% CI. Relevant data values are included in S1 and S2 Data.

To gain insights into the mechanisms underlying dispersal, we investigated whether the onset of group dissolution involved a reduction in the strength of mutual attraction over days. Indeed, earlier work demonstrated in spiderlings of *A*. *labyrinthica* that the formation of aggregates is an active process which relies on a modulation of individual behaviors depending on the number of conspecifics perceived locally: the more conspecifics, the higher the probability of stopping and the lower the probability of leaving an aggregate [5,36]. A simple and efficient means to demonstrate interattraction is to show that the probability of moving for a stopped individual is lower when a conspecific is stopped nearby than when it is alone. At Days 1 and 8 (i.e., before and after dispersal), we introduced either single individuals or pairs of spiderlings to experimental arenas to record either the durations of stops of isolated individuals or the lifetimes of aggregates (the two spiderlings were considered to be aggregated if their interindividual distance was equal or less than 10 mm). We superimposed the paths of two individuals tested alone to determine the lifetime of theoretical aggregates that would have formed randomly without any social interaction (S3 Fig). Irrespective of age, the lifetimes of experimental aggregates were about two times larger than the lifetime of theoretical aggregates (Fig 3A). This showed that the influence of a conspecific on the probability of moving did not change with age.

The mean (±95% CI) proportion of contacts lasting more than one second was greater when spiderlings stopped in the presence of a conspecific (Day 1: 0.52 ± 0.06, *N* = 10; Day 8: 0.56 ± 0.05, *N* = 10) than the values expected if individuals stopped next to each other by chance (Day 1: 0.39 ± 0.03, *N* = 8; Day 8: 0.44 ± 0.03, *N* = 11) (two-way ANOVA: *F*_1,35_ = 18.23, *P* < 0.001). We found no influence of testing day on the proportions of long stops between Day 1 and Day 8 (two-way ANOVA: *F*_1,35_ = 2.35, *P* = 0.13). Overall, both the probabilities of stopping in presence of a sibling and of leaving an aggregate remained constant over time. This means that the decline in aggregation cannot be attributed to a reduction in the strength of mutual attraction.

### An increase in mobility is sufficient to trigger dispersal

We used a numerical model to test the hypothesis that an increase in individual locomotion with age was sufficient to induce dispersal. From data collected for single spiderlings in the present study, we estimated that the probability of moving increased by 1.95 between Day 1 and Days 4–6 and remained constant afterwards (a detailed description of parameter estimation and model implementation is provided in the Supporting Information, S2 and S3 Tables, S4 and S5 Figs). We obtained a close quantitative match in dispersal dynamics between experiments and simulations that included both mutual attraction and the shift in mobility on Day 4 (Fig 3B). Simulations without social interactions (i.e., spiderlings behaving as if alone) confirmed that the initial aggregation required mutual attraction. Similarly, simulations implementing no ontogenic switch in locomotion also showed, as expected, a total suppression of dispersal (Fig 3B). Taken together, these results provided support for our assumption that increased mobility is sufficient to trigger dispersal, which requires no changes in social behaviors.

### Social isolation triggers aggressiveness

**Fig 4:**
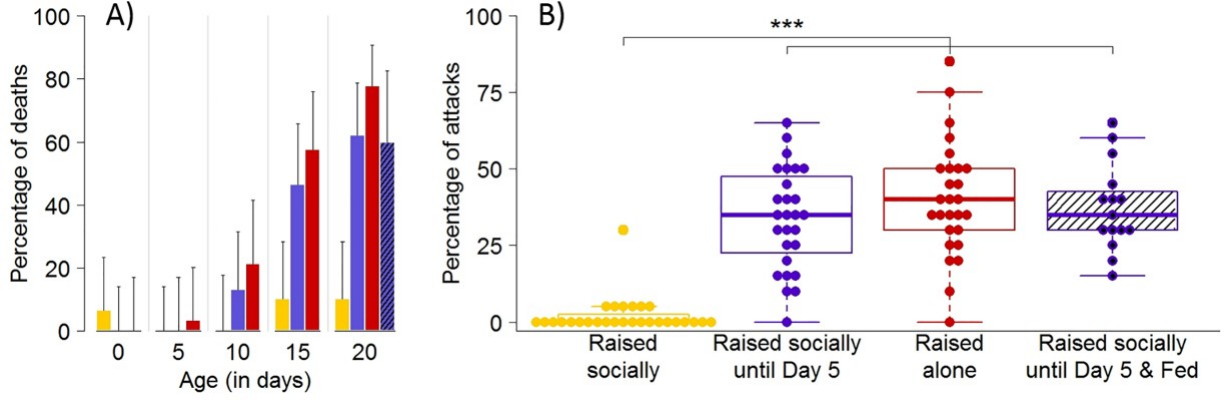
Social isolation triggers cannibalism and aggressiveness. (A) Proportion (± 95% CI) of deaths in pairs of spiderlings as a function of time (days) since emergence. Unfed spiderlings were maintained in groups (yellow bars), in groups for five days before being placed in isolation (blue bars), or alone (red bars) before their introduction to test arenas. The hatched bar gives the rate of cannibalism in pairs of 20-day-old spiderlings raised socially for five days before being placed alone but that were fed three times. Sample size ranged between 24 and 30 replicates for each condition. For statistical analysis, see S4 Table. (B) Boxplots of the proportions of aggressive encounters (bite attempt, effective bite or chasing) between 20-day-old spiderlings raised in groups and unfed (yellow), raised in groups for five days before being placed in isolation (unfed: blue, fed: blue hatched), or maintained alone and unfed (red). A dot gives the proportion of aggressive encounters for each pair. Horizontal line in each box represents the median, and the lower and upper hinges indicate the first and third quartiles. Lower and higher whiskers extend to the most extreme values within 1.5 interquartile ranges from the first and third quartiles respectively. Significant difference is indicated by *** (Dunn's test, *P* < 0.001). Relevant data values are included in S1 Data.

If no loss of tolerance precedes dispersal, what triggers the onset of aggression? Considering that dispersal induced a significant decrease in contact rates among conspecifics, we hypothesized that a reduction in social interactions plays a critical role in the initiation of aggressive interactions. To test our hypothesis, we maintained unfed spiderlings either in isolation or in groups since their emergence from the maternal cocoon (S6 Fig). Pairs of unfamiliar siblings were then introduced in small circular arenas after 5, 10, 15 or 20 days. Over a period of five days, we recorded the occurrence of deaths, which were mainly due to cannibalism, which is the most extreme form of aggression. Cannibalism was rarely observed in pairs of spiderlings raised socially until the test (Fig 4A). This was true even at 20 days, an age at which natural dispersal has long occurred. In contrast, the frequency of cannibalism increased with the time spent in isolation for siblings that experienced no social interactions since hatching. Similarly, individuals maintained socially for five days before being placed alone also displayed an increase in the rate of cannibalism with the time spent in isolation (Fig 4A).

An absence of cannibalism does not necessarily imply the existence of peaceful interactions, as aggression in spiders does not necessarily escalate to cannibalism. We thus needed to confirm that spiders maintained in groups behaved less aggressively than spiders that experienced social isolation. Using video recordings of experimental arenas, we observed the first 20 encounters in each pair to compute the proportion of aggressive interactions (bite attempt, effective bite or chasing) at Day 20. Spiderlings that were maintained in isolation displayed more aggressive interactions (about 40%) than spiders raised in groups (about 2%) (Fig 4B, S1 Movie) (Kruskal–Wallis test: W_3_ = 54.28, *P* < 0.001). All these results indicated that social isolation triggered the onset of aggressiveness and that the rate of cannibalism gradually increased with time spent alone.

### Feeding does not suppress the impact of social isolation on cannibalism

Under the assumption that starvation promotes aggressiveness [37], we explored the influence of nutritional state on the rate of cannibalism and aggressiveness. Indeed, it could be argued that isolated spiderlings incurred more energetic costs than conspecifics maintained in groups and that the difference in body condition between spiderlings raised alone or socially could explain, at least partly, their differences in aggressiveness. Silk-laying represents a possible important source of energetic costs as the production of silk has important metabolic and immune consequences [38]. In the spider *Agelena limbata*, for instance, the cost of web construction requires up to 20 times the daily maintenance energy of an adult [39]. Because spiderlings laid silk on the bottom of the breeding dishes, it was conceivable that individuals in groups shared the investment in silk deposition and experienced reduced costs in comparison to single individuals. Spiderlings fed with one fruit fly per week showed a similar level of aggression and cannibalism than unfed individuals after 20 days of isolation (Fig 4, Dunn's test: Z = −0.43, *P* = 0.34). This indicates that a reduction in the level of starvation cannot compensate the impact of social isolation on aggressiveness.

### Dispersal leads to social isolation and cannibalism

**Fig 5:**
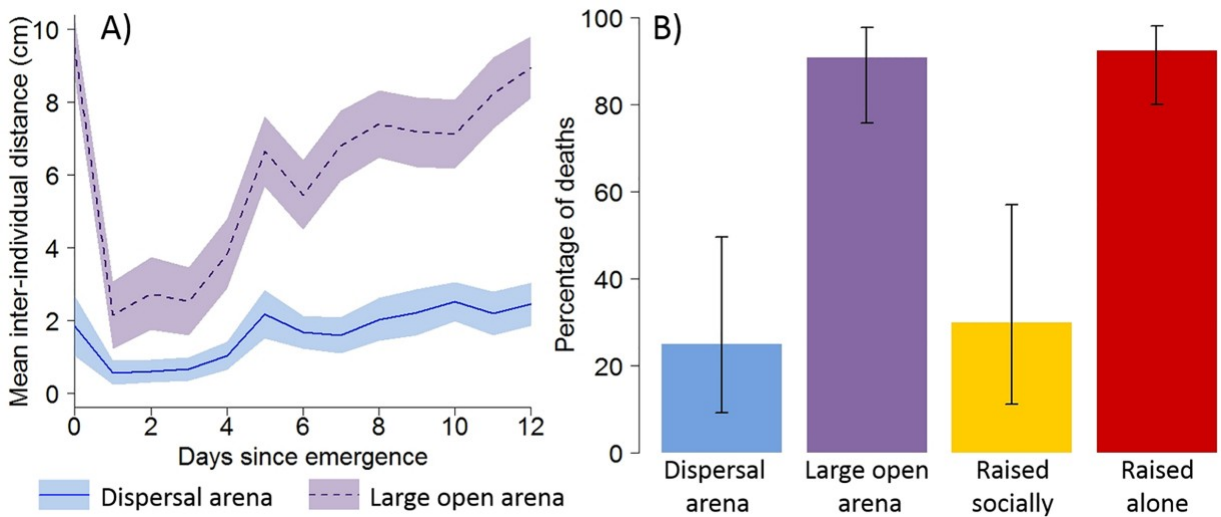
A reduction in social contacts after dispersal results in increased cannibalism. A) Mean interindividual distance as a function of days in groups of four spiderlings introduced to dispersal arenas (diameter = 8 cm) or to large open arenas (diameter = 29 cm). Colored bands are 95% CI. B) Proportion (± 95% CI) of deaths in pairs of unfed spiderlings that were maintained for 17 days in groups of four individuals in dispersal arenas (*N* = 12), in groups of four in large open arenas (*N* = 22), raised socially (*N* = 10) or alone (*N* = 27) in breeding dishes before their introduction to test arenas to record cannibalism. Relevant data values are included in S1 Data.

No cannibalism was observed in dispersal assays because the presence of walls prevented full dispersal, which resulted in the maintenance of residual interactions after the onset of dispersal. Indeed, spiderlings still spent around 10%–15% of their time in close proximity (<6 mm) to a conspecific after Day 9 (S7 Fig). To further establish the close relationship between dispersal, social isolation, and cannibalism, we introduced groups of four spiderlings to large open arenas (diameter: 29 cm) for two weeks. The dynamics of dispersal were similar in both types of arenas, but as expected, spiderlings dispersed further apart in large arenas than in smaller dispersal arenas (Fig 5A). After 17 days, we found that spiderlings from the large open arenas showed a rate of cannibalism matching the score obtained for spiderlings maintained in isolation for two weeks and much larger than the rate measured in spiderlings maintained socially in breeding dishes or in dispersal arenas (Fig 5B).

### Early social experience influences aggressiveness toward a prey

**Fig 6:**
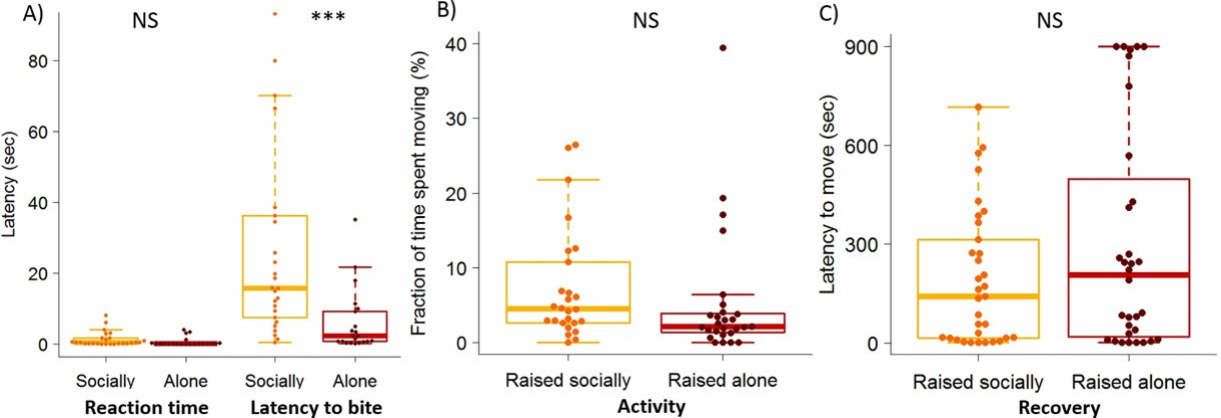
Social isolation influences aggressiveness toward a prey but not the activity level nor the latency to recover after an aversive stimulation. (A) Reaction time and latency to bite a prey introduced to a test arena containing a single spiderling raised socially (*N* = 22) or in isolation (*N* = 21) for 15 days before the test. (B) Fraction of time spent moving by single spiderlings in an open arena (diameter: 55 mm) for 30 min after they were raised alone (N = 27) or in groups (*N* = 26) for 15 days. (C) Latencies to resume activity after being stimulated with an air puff for individuals raised socially (*N* = 33) or alone (*N* = 32). In each box plot, a dot indicates the score of each spiderling. Horizontal line in each box represents the median, and the lower and upper hinges indicate the first and third quartiles. Lower and higher whiskers extend to the most extreme values within 1.5 interquartile ranges from the first and third quartiles respectively. Significant difference is indicated by *** (*t* test, *P* < 0.001), NS indicates non-significant differences. Relevant data values are included in S1 Data.

We next determined whether social isolation influenced the level of aggressiveness toward a prey. No difference in the reaction time (time elapsed before the spider moved after the prey was introduced) was demonstrated between spiderlings raised alone (mean ± 95% CI = 0.66 ± 0.56, *N* = 21) or socially (mean ± 95% CI = 1.31 ± 0.93, *N* = 22) (*t* test: *t*_41_ = 1.71, *P* = 0.09). Interestingly, the latency before the first bite was almost five times shorter for spiderlings raised alone (mean ± 95% CI = 6.12 ± 4.09, *N* = 21) than for those raised socially (mean ± 95% CI = 28.05 ± 12.55, *N* = 22) (*t* test: *t*_41_ = 4.45, *P* < 0.001) (Fig 6A). Importantly, all spiderlings, raised in isolation or in groups, seized the prey in less than 3 min. Social experience had no detectable influence on the level of activity measured as the fraction of time spent moving in an open arena during 30 min (*t* test: *t*_51_ = 1.56, *P* = 0.12) (Fig 6B). Also, we found no difference in the latency to resume movement after receiving an aversive stimulus (air puff) between spiderlings that experienced social interactions (mean ± 95% CI = 194.97 ± 72.85, *N* = 33) or not (mean ± 95% CI = 303.5 ± 122.25, *N* = 32) (*t* test: *t*_63_ = 1.13, *P* = 0.26) (Fig 6C). Altogether, these results provided lines of evidence that the early social context influenced aggressiveness, at least toward a prey, but not the general level of activity.

### Social isolation impairs the response to social cues

One hypothesis to account for an effect of isolation on social interactions implies communication. Indeed, an increase in aggression between siblings might imply that social deprivation altered the way individuals perceived conspecifics. Because communication involves an emitter and a receiver, we tested whether social deprivation impacted the emission of signals and/or the response to social cues in spiderlings. We first examined whether isolation modified the nature of signals produced by spiders. The regulation of many social activities in spiders relies on chemical compounds bound to the cuticle [40]. Hydrocarbons are an important class of cuticular compounds that have been repeatedly shown to play major roles in communication in arthropods. Several factors including age, sex, nutritional status, social context and fluctuations in abiotic conditions are known to cause qualitative and quantitative changes in the blend of cuticular hydrocarbons. Earlier work in spiders revealed the existence of substantial differences in cuticular profiles before and after molting events associated to increased aggressiveness [41,42]. We used gas chromatography to characterize the chemical signatures of spiderlings that experienced isolation compared with those reared socially. Our analysis revealed quantitative variations in the cuticular profiles of spiderlings from distinct cocoons but we found no consistent differences between siblings raised socially or alone (S5 Table, S8 and S9 Figs). This suggested that dissimilarities in the signatures in hydrocarbons were not responsible for differences in aggressiveness in pairs of spiderlings exposed to different social contexts. We next examined whether social deprivation altered the processing of cues emitted by conspecifics. On Day 20, we paired one spiderling raised socially for 5 days and then alone for 15 days with one sibling raised socially for 20 days. Spiderlings from each treatment were individually marked with paint and introduced to a small arena for five days. About 40% of interactions were aggressive and spiderlings raised alone initiated almost all attacks (85%, χ^2^_17_ = 60.34, *P* < 0.001). After five days, 45% of spiderlings raised in groups were cannibalized against 10% of individuals that experienced isolation (χ^2^_1_ = 4.45, *P* = 0.035); relevant data values are included in S1 Data. This provided evidence that isolation impacted the way spiderlings responded to social cues.

## Discussion

**Fig 7:**
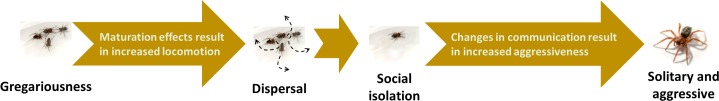
Schematic summary of the causal relationship between dispersal, social isolation and aggression. A maturational increase in mobility in gregarious spiderlings triggers dispersal that results in social isolation, which, in turn, elicits aggression due to changes in the way individuals responded to signals from conspecifics.

In this study, we investigated the causal relationship between the onset of dispersal and aggression in spiderlings of a solitary species. The main findings of our study are summarized in Fig 7.

### Gregariousness is a plastic trait

After an initial phase of intense aggregation of about five days, spiderlings progressively dispersed (Figs 1–3). Our results provided evidence that an ontogenic shift in locomotion, which was shown to be independent of the social context, was likely sufficient to trigger dispersal in spiderlings. In spiders, the duration of the gregarious phase is a highly plastic trait that notably depends on food availability: a high density of prey typically delays the onset of dispersal and reduces the level of aggression [25, 26, 33, 34, 43]. It is generally agreed that such extended social tolerance results from a better nutritional state granted by the reduction in competition with regards to food supply [27, 44]. We introduce here an additional and non-mutually exclusive hypothesis. In arthropods, hunger level is well known to control the locomotion of individuals, which generally increase their movement upon starvation [45]. In the spiders *Hogna helluo* and *Dolomedes triton*, the locomotory activity is influenced by the feeding regime and food-limited individuals travel further than well-fed conspecifics [46, 47]. In arachnids, starvation also lessens the affinity for silk and increases the likelihood of moving on the web periphery [48–50]. Altogether, this indicates that food availability influences movement patterns in spiders. We propose that a high density of prey limits the need to move over long distances for hunting and increases the frequency of social interactions [44]. The exposure to social interactions might favor the maintenance of amicable interactions among otherwise more aggressive spiders. In *Coelotes terrestris*, for example, the confinement of spiderlings in restricted enclosures with abundant prey can drastically reduce the rate of cannibalism and can even lead to the formation of incipient societies with reproduction among adults [27]. Therefore, food availability might shape the level of tolerance directly by its action on body condition and indirectly via social exposure.

### Social contacts are necessary for social tolerance

In line with our previous argument, we demonstrated that spiderlings that experienced social isolation were much more aggressive toward siblings than those reared socially (Figs 4 and 5). This suggested that social intolerance was a consequence, not a cause, of dispersal. In a diversity of taxa, the frequency of social interactions plays an important role in the modulation of social behaviors. For instance, crowding associated with an increase in tactile stimulation induces cannibalism in the larval tiger salamander *Ambystoma tigrinum* [51] or, in contrast, elicits behavioral gregariousness in the otherwise solitary locust *Schistocerca gregaria* [52]. Here, we showed that social contacts are required to preserve social tolerance. The need for extensive social interactions for the maintenance of tolerance is possibly an overlooked and general principle that can reconcile many observations in spiders, with the possible exception of spiderlings of the genus *Latrodectus* that behave aggressively before dispersal [53]. For instance, species typically show a transient gregarious phase immediately upon hatching after they experience intense contacts in the cocoon, where they are densely packed, for several consecutive days or months. Also, the female in subsocial species provisions her offspring by sharing prey or laying trophic eggs [54, 55]. In doing so, the mother fosters prolonged interactions among spiderlings and this likely contributes to the preservation of social tolerance for relatively longer periods of time in comparison to species where maternal care is absent.

The indication that extended social contacts are required for the maintenance of social tolerance in spiderlings invites questions regarding the underlying mechanisms. The impact of social isolation on physiological and behavioral traits has been documented in vertebrates and also in invertebrates, albeit to a lower extent [56,57]. A deprivation of social contacts is notably known to influence the expression of aggressive behaviors such as in the crayfish *Cherax destructor* [58] or in the field cricket *Gryllus bimaculatus* [59,60]. Here, we found that individuals raised alone attacked prey more rapidly than siblings maintained in groups and, also, that socially deprived spiderlings initiated most aggression when paired with a sibling raised socially (Figs 5 and 6). This suggests that repeated contacts among spiderlings are necessary to preserve the ability to process and interpret conspecifics' cues, possibly learnt during early ontogeny. In spiders, the expression of tolerant behaviors requires the processing of chemical information as evidenced, for example, in the wolf spider *Geolycosa turricola* where spiderlings with impaired chemoreception perform more aggression than do untreated individuals [61]. In the highly integrated colonies of ants, the social isolation of workers impairs nestmate recognition because of a partial loss of the internal template used to discriminate nestmates from non-nestmates [62,63]. Here, the analysis of the chemical signatures of spiderlings revealed similar profiles between spiderlings raised alone or in groups, which strongly suggested that social isolation influenced the processing, not the emission, of social cues. Social isolation apparently induced a loss of the ability to respond amicably to cuticular compounds carried by siblings. This could also partly explain the general rise in aggression following the first molt after dispersal, which coincides with massive changes in the cuticular profiles [41,43]. A mismatch in the chemical signatures of siblings from different developmental stages is thus expected to elicit increased agonistic behaviors.

A high level of aggressiveness is considered to be the default condition in the field cricket *Gryllus bimaculatus* and the low aggression reported in grouped individuals is a manifestation of social subjugation resulting from the expression of loser effects [59]. In spiderlings, no aggressive interactions were observed before dispersal and the contribution of loser effects is unlikely to explain the high tolerance observed in gregarious spiderlings. So far, our preferred hypothesis involves habituation to social cues that first starts in the cocoon when spiderlings are densely packed before hatching and that continues during the gregarious phase. The reduction of the rate of social interactions after dispersal possibly induces individuals to forget social cues and initiate aggression.

### Transitions to permanent sociality

One main route to permanent sociality in spiders involves the extension of the period of juvenile tolerance [64]. Our study showed that a decline in the rate of social interactions triggers the loss of tolerance in solitary spiderlings. Comparative work would now be particularly recommended to investigate how juveniles of social spiders respond to social isolation. Our prediction is that social spiders are less sensitive to social deprivation than phylogenetically related species of solitary spiders (e.g., *A*. *labyrinthica* versus *A*. *consociata*). Indeed, tolerance apparently shows less plasticity in social than in solitary species. For example, adults of the social species *Mallos gregalis* starved to death rather than resorting to cannibalism [65] while a reduction of prey availability incites cannibalism in solitary spiders [66]. Empirical support to our prediction would raise questions about the proximal changes in the processing of social cues associated with the transitions to permanent sociality in spiders and that lead to tolerance towards conspecifics. Interesting avenues for reflection are provided by studies in halictine bees, which also display a diversity of social structures and in which evolutionary transitions between solitary and social living have been showed to be accompanied by changes in the sensory systems involved in signal perception [67].

### Conclusion

Spiders are key actors in the regulation of terrestrial ecosystems [80]. Among the many facets of their biology that are still poorly understood, their transient gregariousness only received little attention. This is surprising given that this life-history trait is shared by most, if not all, species of spiders. Our results revealed a counter-intuitive mechanism to explain such a social transition: the increased aggression towards conspecifics is a consequence, not a cause, of dispersal in spiderlings. We further demonstrated the fundamental role of extended social contacts on the maintenance of tolerance and that the onset of aggression relies on a shift in the way spiderlings processed the signals emitted by conspecifics. Given the robustness of the mechanisms involved in aggregation, our findings are expected to be valid for the vast majority of solitary spiders. Overall, our study offered new insights into the mechanisms of dispersal in a species with transient sociality and opens new avenues for understanding the routes to permanent sociality.

## Materials and methods

### Ethics statement

This work complies with the current laws in France on the use of invertebrates in research.

### Model species

*A*. *labyrinthica* (Agelenidae, Clerck 1758) is a solitary web-building spider widely distributed across southern and central Europe. Females usually lay one to three cocoons containing an average of 80–120 eggs in late summer. Hatching occurs inside the cocoon after a period of incubation of about 20 days. At this stage, larvae show little mobility and obtain their nutrients from egg yolk. This first instar lasts one week and ends with a first moult in the cocoon (second instar). Spiderlings then enter a winter diapause until early spring when they emerge from the maternal cocoon and remain gregarious for a few days prior dispersal [5]. Second-instar spiderlings are mobile, produce silk and display hunting behaviors toward prey [41]. The average body length (prosoma+opisthosoma) of 2nd-instar spiderlings is 2.5 ± 0.29 mm (mean ± standard deviation, *N* = 60) [5]. We used a total of 46 cocoons collected in southwest France in 2016, 2017, and 2018 (13 cocoons were collected near Marquefave: 43°19'N; 1°15'E, 13 cocoons near Lézat-sur-Lèze: 43°16''N; 1°20'E, 6 cocoons from La Roquille: 44°47'N; 0°14'E, 8 cocoons near Duras: 44°40'N; 0°11'E, and 6 cocoons from Ramonville Saint Agne: 43°32'N; 1°29'E). After field collection, cocoons were maintained in a cooled incubator at 4°C in darkness to simulate winter diapause until the beginning of experiments. When needed, cocoons were removed from the incubator and gently opened with callipers to extract spiderlings. Opening the cocoons rather than letting spiderlings emerge spontaneously guaranteed that siblings all experienced the same social context before being used in behavioral assays. The cocoon used in this study contained 103 ± 37 spiderlings (mean ± standard deviation) (median = 97; minimum = 10; maximum = 206; *N* = 46). Hereafter, we referred to the day of opening as the day of emergence or Day 0. Only 2nd-instar spiderlings were used. Spiderlings tested in groups were always siblings and never fed during experiments unless otherwise stated.

### Onset of dispersal

These experiments aimed at precisely monitoring the onset of dispersal in groups of spiderlings. Five cocoons (two from Marquefave, two from la Roquille, and one from Duras) maintained in the incubator since October 4 2017 were opened between January 4 and 6 2018. Spiderlings from each cocoon were randomly assigned to one of three treatments:

- groups of four individuals were introduced to a dispersal arena (see below) on the day of emergence (Day 0) (*N* = 10),
- groups of eight spiderlings were maintained in a breeding dish (transparent plastic Petri dish, diameter: 55 mm, height: 15 mm) for five days. On Day 5, the groups of eight spiderlings were separated in two groups of four spiderlings that were introduced to dispersal arenas (*N* = 10),
- groups of eight spiderlings were maintained in a breeding dish for 10 days. On Day 10, the groups of eight spiderlings were separated in groups of four spiderlings that were introduced to the dispersal arena (*N* = 9).

The breeding dishes were maintained in a climate-controlled room (24 ± 1°C, 35% RH) under a 12:12-h light:dark cycle (light at 8:00 and dark at 20:00).

The dispersal arenas consisted of transparent plastic dishes (diameter: 80 mm, height: 26 mm) partitioned with transparent walls into four compartments of equal surface area. The ends of the walls were removed (diameter: 20 mm, height: 3mm) at the center of the arena to allow spiderlings to move between compartments (Fig 1). The arena was covered with a lid to prevent spiderlings from escaping. Four holes (diameter: 7 mm) were drilled in the lid (one hole above each compartment) for introducing spiders to the arena. One spider was introduced to each compartment, and each hole was then covered with a transparent glass microscope slide. In offering a more complex environment than an open arena, an arena partitioned into four quadrants allowed each spider the opportunity to settle in a quadrant. Also, the junctions between the inner and outer walls formed corners that are generally attractive for arthropods, including spiders. The formation of an aggregate comprising all individuals in a partitioned arena thus lent additional support for the contribution of social interactions in clustering. Partitioned arenas were also preferred for logistical reasons as they minimized the use of lab space. To reduce any spatial heterogeneity that might influence behaviors, we placed arenas in a white box (650 x 650 x 650 mm) in a climate-controlled room (24 ± 1°C, 35% RH) under a 12:12 h light:dark cycle (light at 8:00 and dark at 20:00). Arenas and breeding dishes were watered every two days with an atomizer. Each arena was photographed three times a day at 9:00, 14:00, and 18:00 for 17 consecutive days. From these pictures, we computed the mean nearest-neighbor distance between spiderlings considering the presence of walls (see inset Fig 1) (i.e., an individual that moved from one compartment to another necessarily went through the center of the arena). The three measures of the mean nearest-neighbor distances obtained every day were then averaged to estimate the daily level of aggregation. We next examined to what extent the level of aggregation observed in the experiments differed from the level of aggregation expected under the hypothesis that individuals did not interact (i.e., random distribution of individuals in the arena). We employed the data from the groups introduced to dispersal arenas at Day 0 and surveyed until Day 17. For each day, we used the coordinates of four individuals that were drawn randomly from four (out of 10) different arenas to compute the mean nearest-neighbor distance. This procedure was repeated 500 times to obtain the random level of aggregation. The comparison between groups introduced at Day 0 and groups introduced at Days 5 and 10 allowed us to disentangle the possible role of novelty, habituation or the presence of silk from the influence of maturation on the initiation of dispersal.

### Mobility patterns

This experiment aimed to characterize the changes in mobility during ontogeny. Ten cocoons (three from Marquefave, two from la Roquille, three from Duras and two from Lézat-sur-Lèze) maintained in the incubator since October 8 2017 were opened between February 13 and 18 2018. From each cocoon, single spiderlings (*N* = 14) or groups of four individuals (*N* = 14) were introduced to dispersal arenas. Single individuals were placed randomly in one compartment. We photographed the arenas every 10 min for 12 consecutive days with a camera (Canon EOS 50D) placed under arenas. No water was provided during these experiments to prevent the formation of condensation, which would have been detrimental for image analysis. Note that we evidenced no influence of the absence of water on the dynamics of dispersal (S10 Fig).

We used the function Analyze Particles of the software ImageJ 5.1 to extract automatically the coordinates of each individual from the pictures of dispersal arenas (every picture was then checked by eye to control the accuracy of detection). For single spiders, we calculated the distance covered between two successive photographs considering the presence of walls. In groups, spiderlings were not marked so it was not possible to identify each spider between consecutive pictures and to determine to what extent each individual moved between two timesteps. We estimated the distance covered by each spiderling in groups by assuming that it minimized its displacement between two time steps and we averaged the four measures to obtain the mean individual distance covered in groups. We acknowledge that our approach was rather conservative and underestimated the actual distance covered by spiderlings. However, our goal was not to obtain an accurate measure of the distance covered by individuals but we rather aim to provide a qualitative proxy to compare the mobility between spiders tested alone or socially.

In addition, we computed an index of activity over 24-h period for the calibration of the numerical model (see below). We considered that a spider moved if its position changed by one body length or more between two consecutive photographs. The threshold for movement was set to 3 mm, which corresponds approximately to one body length. For isolated spiderlings, we calculated for every 24-h period the proportion of consecutive pairs of timesteps where the individual moved a minimum of 3 mm. For groups, we proceeded similarly in considering the number of spiders in each arena that moved between two consecutive timesteps. The proportions were then averaged across replicates for spiderlings tested alone or in groups.

### Synchronization in the onset of activity

To confirm that social interactions were not involved in the onset of activity, we examined whether the increase occurred simultaneously for siblings tested alone or in groups. Comparing siblings allowed us to limit possible confounding effects due to maternal and/or genetic effects that might impact the onset of activity. From the pictures of dispersal arenas containing one or four spiders, we set the threshold for movement to 3 mm and we determined whether individuals moved between two consecutive time steps (10 min). Starting at 8:00 of the second day (i.e., after spiderlings aggregated), we considered that the onset of mobility occurred at the end of the 6-h interval where a spider was first observed performing nine or more movements (which represent 25% or more 10-min time steps of a 6-h interval). By doing so, we found, for example, that the onset of mobility for the isolated individual of the early cocoon represented on Fig 2 occurred at Day 3 plus 14 h. For groups, to avoid sampling effects (i.e., larger probability of detecting a move in groups of four spiderlings than for single individuals), we selected randomly one spiderling at each time step and we determined whether it moved between two consecutive pictures. For example, we determined that mobility increased at Day 4 plus 14 h for the groups of four spiderlings of the early cocoon represented on Fig 2. We then used a Spearman rank correlation test to determine whether the timings of the onset of mobility observed in siblings tested alone or in groups were correlated. A significant correlation revealed that the social context did not influence the increase in activity.

### Contact rate

The movement of spiders was constrained by the presence of the outer walls of arenas, which prevented full dispersal. As a consequence, social interactions between spiderlings likely persisted over time, even after the decline of aggregation and the onset of dispersal. We assessed this effect by computing for each day and for each individual the fraction of pictures where the individual was at less than 6 mm from at least one conspecific. In spiders, chemical recognition requires a contact and involves the perception of cuticular compounds via chemoreceptors present on the distal segments of legs [68]. A threshold of 6 mm was used because this is approximately the distance required to touch each other with the legs in 2nd-instar spiderlings of *A*. *labyrinthica* (body length: 2.5 mm).

### Interattraction

To examine whether the strength of interattraction declined over time, we used data on spiderlings from three cocoons (collected in Marquefave) maintained in the incubator since October 10 2016 and opened on January 24 2017. Groups of four siblings (*N* = 30) were placed in breeding dishes (diameter: 55 mm, height: 15 mm) in a climate-controlled room (24 ± 1°C, 35% RH) under a 12:12-h light:dark cycle (light at 8:00 and dark at 20:00). Breeding dishes were watered every two days with an atomizer. On the day of testing, spiderlings were removed from the breeding dishes and introduced alone or in pairs (from the same breeding dish) to white PVC circular arenas (diameter: 44 mm, height: 3mm). The arenas were placed on white paper and covered with a glass plate to prevent spiders from escaping. To avoid any spatial heterogeneity that might bias behaviors, the arenas were placed in a white box (650 x 650 x 650 mm) with a central hole on top for recordings. Trials lasted 24 h and were recorded continuously with a video camera (Sony Handycam HDR-XR350). During night, arenas were lighted with a red bulb. Individuals were tested on Day 1 (*N* = 10 pairs, 8 isolated) and Day 8 (*N* = 10 pairs, 8 isolated) (i.e., before and after dispersal).

Earlier studies showed that the contribution of interattraction to the formation of aggregates relies on a modulation of individuals' behaviors that depends on the presence of other individuals in their vicinity: the probabilities of stopping and resting times are both higher when the numbers of conspecifics were larger [5, 69, 70]. We thus examined both facets to determine whether interattraction declined over age.

First, we compared the influence of conspecifics on the probability of moving for a spiderling that was stopped alone or in contact to another spider. Each 24h-movie was analyzed with the tracking software SwisTrack (v4.0) using the “Nearest Neighbour Tracking” method at a rate of 2.08 frames per second to extract automatically the coordinates of spiderlings in arenas. For each pair, we calculated the lifetimes of aggregates by considering that two individuals were aggregated if their interindividual distance was less than 10 mm for more than one second [5]. We deliberately used a larger criterion than the one used to estimate the probability of stopping and the encounter rates (6 mm) because a too stringent criterion could be detrimental for the estimation of the durations of long stops as stopped spiders sometime made very small movements. Henceforth, we refer to these pairs as real pairs. To assess the strength of interattraction, we needed to compare the values of the lifetimes obtained in real pairs against the lifetimes of aggregates that would have formed randomly without any social interactions (i.e., the duration of an aggregate formed by two spiders that would have stopped by chance in close proximity but that would not modulate the duration of their stops as a function of the presence of a conspecific). To obtain the distribution of the lifetimes of aggregates under the null hypothesis that spiderlings did not interact, we superimposed the coordinates of spiders from the same cocoon that have been tested alone in arenas (*N* = 14) to form virtual pairs (S3 Fig). We used the same criteria as for real pairs to determine the lifetimes of aggregates in the virtual pairs. The comparison between the lifetimes of aggregates in virtual and real pairs revealed the contribution of social interactions to aggregation. For each day, we compared the survival curves of aggregates' lifetimes between real and virtual pairs using a Cox proportional hazard model (function *coxph* implemented in the package *Survival* in R) (Fig 3). The Cox proportional hazard model is a semiparametric survival model that necessitates no specification of its baseline hazard function. The hazard ratio estimates the difference in the hazard rate (i.e., instant probability for the event of occurring at time *t*) between two samples [71]. We used the hazard ratio associated with its 95% CI as a proxy to quantify the strength of interattraction. A hazard ratio of 1 indicates similar lifetimes between real and virtual aggregates. A ratio greater than 1 reveals interattraction (longer durations for real than for virtual aggregates, i.e., lower probability of leaving the aggregate). Ratio less than 1 would indicate active avoidance (i.e., higher probability of leaving an aggregate than expected by chance).

In addition to the quantification of the influence of conspecifics on stop durations, we also determined to what extent the probability of stopping was influenced by the presence of another spider. For each pair, we divided the number of contacts that lasted more than one second by the total number of contacts (a contact occurred if the interindividual distance was equal or less than six mm, see above). This proportion was used as a proxy to estimate the influence of the presence of a conspecific on the probabilities of stopping. We compared the proportions calculated for real and virtual pairs at Days 1 and 8 with a two-way ANOVA.

### Numerical model

We developed a spatially explicit agent-based model written in Java. Our aim with the model was to explore the contribution of social interactions and mobility to the dynamics of aggregation and dispersal observed in our behavioral assays. To this end, we first ran simulations implementing the ontogenic shift in mobility (i.e., the probability of moving varied with age, see below) but not the social interactions (i.e., spiderlings behaved as if they were alone in the arena) to estimate the level of aggregation expected by chance. Next, we did the opposite in running simulations that implemented interattraction (i.e., a modulation of individual behaviors depending on the presence of conspecifics, see below) but not the ontogenic change in mobility. Finally, we ran a full model implementing both interattraction and the changes in mobility with age. The comparison of the collective dynamics between these different conditions allowed us to determine whether the ontogenic change in mobility was sufficient to explain the onset of dispersal in our experiments.

A description of the behavioral algorithm is provided in S4 Fig and all the probabilities and parameters used in our model are summarized in S2 and S3 Tables and in the text below. We explain below how the values implemented in the model were obtained from experiments. More technical details can be found in our earlier studies [5,36,72]. Basically, our model implemented the movement of an individual in the arena and the rules governing social interactions.

#### Modelling individual movement

The modelling of individual paths requires to distinguish paths occurring at the edge of the arena (thigmotaxis) or in the centre of the arena [72]. We considered that a spider displayed wall-following behavior when it was less than two body lengths (5 mm) from the wall [5]. When following the walls of the arena, we considered that spiderlings had a linear movement mode with a constant probability per unit time of leaving the edge and entering the central zone of the arena. Far from the walls, the movement of spiders was modelled as a diffusive random walk (for details, see [72]). The spontaneous probability of stopping per unit of time for a moving individual differed as a function of the spatial location (close or far from the walls) [5]. The presence of inner walls added a spatial constraint that was not present in the initial model [5]. Here, we set the probability of stopping at the end of an inner wall to *P*_*w*_ = 0.6 and the probability of stopping at the corner between an inner wall and the outer wall to *P*_*c*_ = 0.4. The probability of moving for spiders stopped alone differed from the study of Mougenot et al. [5], which focused on aggregation dynamics for three h while here we aimed at modelling aggregation for 11 days. We thus needed to characterize the durations of stops over longer time scales. We used the durations of stops measured for single individuals on Day 1 (see above, **Interattraction**) to compute the individual probabilities of moving. The survival curves of the lifetimes of stops for single individuals were characterized by a fast decay followed by a slow decay (Fig 3A). Such distribution indicated that the probability of moving decreased with the time spent stopped. In line with our earlier work on *A*. *labyrinthica* [5] and other species such as the cockroach *Blattella germanica* [69, 70] and the fruit fly *Drosophila melanogaster* [73]), we fitted the fraction of isolated individuals still stopped as a function of time *t* with the function:

$$F(t)=\frac{e^{-\delta t}}{{\left(1+\frac{t}{\beta }\right)}^{\alpha }}\mathrm{where}\;\alpha ,\;\beta \;\mathrm{and}\;\delta \;\mathrm{are}\;\mathrm{constants}$$ (1)

From Eq 1 the probability per unit of time of moving is given by:

$$P_{leave}=\delta +\frac{\alpha }{\beta +t}$$ (2)

We performed a nonlinear regression using the function *optim* from the package *stats* in R to fit the experimental survival curve with Function 1. A good fit was obtained for *α* = 0.48 and *β* = 0.74. The value of *α* informs on how quickly a stopped individual initiated a move and the value of *β* modulates the influence of the time spent stopped on the probability of moving (a high value of *β* implies that an individual has to stay stopped for a long before its increased staying time significantly influences its probability of moving). The addition of a constant *δ* (here, set to 10^−6^) allowed the probability per unit of time of moving to plateau for long resting times [70].

We needed to model the observed change in mobility over time. Indeed, the experimental dynamics revealed a substantial increase in mobility around Day 4 (see results in main text, Figs 1, 2 and 5A). An increase in mobility was associated with a reduction in the duration of stops, which can be modelled by an increase in the value of *α* (Eq 2): the larger *α*, the greater the probability of moving. It was not meaningful to use the lifetimes measured on Day 8 of single individuals introduced alone in experimental arenas (as we did to estimate the probability of moving on Day 1). Indeed, these experiments to measure the durations of stops on Day 8 were performed in an arena without silk and the quantity of silk is known to influence resting time in spiderlings [74]. Instead, we ran simulations with single individuals to determine what increase in *α* at Day 4 was necessary to reproduce the experimental increase in mobility observed in isolated spiderlings. A satisfactory agreement between the experimental and simulated indices of activity (percentage of time moving) measured over days was obtained for 1.95-fold increase in the value of *α* at Day 4 (S5A Fig).

#### Modelling social interactions

Modelling social interactions in the context of aggregation required to characterize the influence of conspecifics on the individual probabilities of moving for a stopped individual and of stopping for a mobile individual. In the present model, the probabilities of stopping as a function of the number of spiderlings and the probabilities of moving after a collision were borrowed from our earlier study on 2nd-instar spiderlings of *A*. *labyrinthica* [5] (S2 and S3 Tables). It was not possible however to use the published values of the probabilities of leaving aggregates as these parameters derived from 3-h tests while the experiments here lasted several days. We used the lifetimes of aggregates measured for pairs of spiderlings on Day 1 (see above, **Interattraction**) to compute the individual probabilities of leaving an aggregate. The survival curves of the lifetimes of aggregates were characterized by a fast decay followed by a slow decay (Fig 3A). We used Eq 1 to fit the fraction of aggregates still intact (i.e., no individual left the aggregate) using the same value of *β* than for single individuals (a similar value of *β* facilitates the comparison of the probability of leaving the stopped state for isolated or aggregated spiders as only the value of *α* changed between the two conditions, see [69]). The best fit for pairs was obtained for *α* = 0.57. The value of *α* for pairs gave the rate of dissolution of an aggregate but provided no information on the individual probability of leaving it. The probability for an individual of leaving a pair was thus obtained by dividing the value of *α* by 2 (i.e., the number of individuals in the aggregate) (see [69]). The lower value of *α* calculated for an individual in a pair, which equaled 0.285 (i.e., 0.57/2), than for an individual alone (*α* = 0.48, see above) showed that the probability of moving for a stopped individual decreased in the presence of a conspecific, thereby revealing interattraction [36].

In our experiments, we did not record the lifetimes of aggregates of three and four spiderlings, which would have been required to compute experimentally the probabilities of leaving aggregates of three or four conspecifics over long durations. Instead, we used the model to explore what values of *α* for individuals in groups of three or four spiders produced a quantitative agreement in the level of aggregation between simulations and experiments on Day 2 (S5B Fig). A satisfactory agreement was obtained by dividing the values of *α* measured for single individuals by 5 and 10 to compute the individual probabilities of leaving aggregates of three and four, respectively. A stopped individual, either alone or aggregated, could start moving after a collision [36]. We used the probabilities of moving after a collision computed in our earlier work [5] (S3 Table).

At the beginning of a simulation run, each spider was initialized in a moving state at the center of each compartment (one spider per compartment). In total, 1,000 simulations were run for each condition and the time step was 1 s/cycle.

### Social experience and cannibalism

We performed a series of experiments to confirm that social isolation triggered aggressiveness and cannibalism. Between January 2 and 6 2018, we opened 19 cocoons (seven from Marquefave, four from la Roquille, five from Duras and three from Lézat-sur-Lèze) which were maintained in the incubator since October 4 2017. On Day 0, spiderlings were assigned to different rearing conditions (S6A Fig):

1. Spiderlings were maintained in groups of six individuals from Day 0 until being tested either at Day 0 (*N* = 30 pairs), Day 5 (*N* = 30 pairs), Day 10 (*N* = 23 pairs), Day 15 (*N* = 29 pairs), or Day 20 (*N* = 29 pairs).
2. Spiderlings were maintained in groups of six individuals from Day 0 until Day 5. They were tested at Day 0 (*N* = 30 pairs) or Day 5 (*N* = 24 pairs). They were then placed alone in a breeding dish until being tested either at Day 10 (*N* = 30 pairs), Day 15 (*N* = 28 pairs), or Day 20 (*N* = 29 pairs). On Day 20, we also tested spiderlings that were fed with one female fruit fly (*Drosophila melanogaster*) on Day 3, Day 9 and Day 15 (*N* = 15 pairs). Prey remains were removed on days of feeding to minimize disturbance. Almost half of fed spiderlings (25 out of 60) molted before the day of test and were not tested, which confirms that providing spiderlings with prey actually resulted in feeding. Note that molting occurred very rarely in unfed spiderlings (seven spiders out of 586 individuals raised for this experiment, which originated from a single cocoon, which was discarded from the analysis).
3. Spiderlings were maintained alone from Day 0 until the test at Day 0 (*N* = 24 pairs), Day 5 (*N* = 28 pairs), Day 10 (*N* = 28 pairs), Day 15 (*N* = 26 pairs), or Day 20 (*N* = 27 pairs).

For each treatment, spiderlings were maintained in breeding dishes (diameter: 55 mm, height: 15 mm) placed in a climate-controlled room (24 ± 1°C, 35% RH) under a 12:12-h light:dark cycle (light at 8:00 and dark at 20:00). Spiderlings were watered every two days. To ensure that spiderlings from all treatments experienced the same number of manipulations spiderlings from all treatments were introduced to a new breeding dish on Day 5.

Behavioral assays consisted in the introduction of two individuals to a PVC arena (diameter: 22mm, height: 4mm) covered with a glass lid. Individuals tested were siblings but collected from two different breeding dishes (i.e., no familiarity). The arenas were checked daily for five consecutive days to record death events. Spiderlings were tested only once. The rates of cannibalism were compared using pairwise chi-squared tests followed by Holm's corrections to adjust *p*-values for multiple comparisons.

### Social experience and aggressiveness

Because aggressive interactions do not necessarily lead to cannibalism, we needed to characterize more finely the nature of social interactions in spiders exposed to different treatments. Immediately upon the introduction of pairs of spiderlings (*N* = 15 to 29) in arenas on Day 20, we recorded their behaviors during three h with a video camera (Sony Handycam HDR-XR350). For each pair, we then observed the first 20 encounters starting 10 min after the beginning of the test. We determined the fraction of aggressive interactions which include bite attempt (one spider lunges forward to seize its conspecific), effective bite (one spider bites the other) and chasing (one spider moves rapidly toward the other) [41]. One pair that displayed less than five interactions was discarded from the analysis.

In all these trials, spiderlings were tested against an individual from the same treatment and this therefore does not inform us whether spiderlings maintained in isolation or in groups differ in aggressiveness. On Day 20, we thus paired one individual maintained in groups since Day 0 with one individual that was isolated since Day 5 (*N* = 18 pairs) (S6B Fig). The day before the test, spiderlings were briefly (20 seconds) anesthetized with CO_2_ exposure and they were marked with a dot of red or green paint (Edding 750) on the opistosoma. We verified that marking had no effect on mortality rate during cannibalism tests (marked versus unmarked spiders reared alone: χ^2^_1_ = 1.05, *P* = 0.20, N > 14; marked versus unmarked spiders socially: χ^2^_1_ = 0.24, *P* = 0.62, N > 12). On Day 20, the two individuals were introduced to an experimental arena (see above). Immediately upon their introduction, we recorded the behaviors of spiderlings during three h with a video camera (Sony Handycam HDR-XR350). We then determined for the 20 first encounters in each pair the proportion of aggressive interactions and the identity of the initiator. We then monitored for five consecutive days the occurrences of death. The proportions of aggressive encounters were compared between treatments using a Kruskal–Wallis test followed by a post-hoc Dunn's test.

### Dispersal in large arenas and cannibalism

We performed an experiment to examine whether the reduction in the frequency of social interactions with time triggered to the onset of cannibalism. We used spiderlings from nine cocoons (five from Ramonville Saint Agne, four from Lézat-sur-Lèze) placed in the incubator on October 8 2018 and opened between February 4 and 7 2019. Groups of four spiderlings were introduced to a large PVC arena (diameter: 290 mm, height: 10 mm) covered with a glass plate (*N* = 15). We also introduced groups of four spiderlings to dispersal arenas (*N* = 15). The large open arenas and the dispersal arenas were photographed daily at 14:00 for 12 consecutive days. From these pictures, we computed the mean nearest-neighbor distance between spiderlings (see **Mobility patterns** section). In control experiments, spiderlings were maintained in groups of five (*N* = 15) or they were placed alone (*N* = 60) in breeding dishes. Spiderlings were not watered to prevent any disturbances that might have been possibly induced by lifting the lid of the large arena to spray water. We discarded from the analysis the replicates (*N* = 4 to 9) in which mortality was observed. After 17 days, spiderlings were removed from their arenas and introduced in pairs to a PVC arena (diameter: 22 mm, height: 4 mm) covered with a glass lid. We checked daily for five consecutive days to record death events.

### Aggressiveness toward a prey

We aimed to test whether social isolation impacted aggressiveness toward a prey. We used three cocoons (two from Lézat-sur-Lèze, one from Ramonville Saint Agne), maintained in the incubator since October 10 2018 and opened on February 6 2019. Spiderlings were introduced either alone or in groups of five to breeding dishes and watered every two days. The day before the test, two spiders from the same cocoon were introduced to a circular plastic dish (diameter: 25 mm, height: 10 mm) covered with a glass lid to lay silk for 24 h. The day of the test, we removed from the test arena the spiders that laid silk (taking care to preserve web structure), and we introduced a spiderling that was either maintained alone (*N* = 27) or in groups (*N* = 26). After about 60 min of acclimation, we gently removed the lid covering the test arena to introduce one fruit fly (*Drosophila melanogaster*). The experimenter was blind to each spider's treatment. Each trial was recorded with a video camera (JVC EverioR GZ-RX645AE). We then used the video-recordings to measure the reaction time of the spiderling (i.e., the time elapsed before the spider made a first move upon the introduction of the prey to the test arena) and the latency before the first bite that lasted at least three seconds. Spiderlings were then allowed to consume the prey for five to six h before the subsequent experiment (see **Activity level** section). We discarded trials (*N* = 6 and *N* = 4 for spiderlings raised alone and socially, respectively) in which the flies fell down on spiderlings when they were introduced to the test arena. Latencies were compared using *t* tests after logarithmic transformations to meet the assumption of homoscedasticity.

### Activity level

We compared the level of activity between spiderlings that were maintained alone or in groups. Five to six h after the completion of the trials to estimate the aggressiveness toward a prey (see **Aggressiveness toward a prey** section), we introduced spiderlings alone to circular arena (transparent plastic dish: diameter: 55 mm, height: 8 mm) to record their activity. After 15 min of acclimation, we recorded the tracks of each spider using a video camera (Sony Handycam HDR-XR350) for 30 min. The tracks were then analyzed with the tracking software SwisTrack to extract automatically the coordinates of the spiderlings in arenas at a sampling rate of 3 frames per second. We compared the fraction of time spent moving (moving threshold = 0.5 mm) during 30 min between spiderlings raised alone or socially with a *t* test after square root transformation.

### Latency to resume movement after receiving an aversive stimulus

Three cocoons maintained in an incubator since 4 October 2017 were opened on 7 March 2018. Spiderlings were introduced either alone or in groups of six (see above) to breeding dishes and watered every two days. At Day 15, spiderlings were introduced alone in a plastic dish (diameter: 55 mm, height: 15 mm) without lid. We used an air puff as an aversive stimulus [75]. After five min of acclimation, we used a 20 ml syringe to deliver a puff of air at approximately 15 mm from the cephalothorax and with an angle of about 30°. After receiving an air puff, spiderlings responded by huddling and we recorded for each spider the latency to resume movement. The experimenter was blind to each spider's treatment. We compared latencies between spiderlings maintained alone (*N* = 32) or socially (*N* = 33) using a *t* test after square root transformation.

### Chemical analysis

We aimed at examining whether social isolation influenced the profiles of cuticular hydrocarbons of spiderlings. We used spiderlings from three cocoons (two from Duras, one from Lézat-sur-Lèze) placed in the incubator on 4 October 2017 and opened between 3 and 5 March 2018. On Day 22, we killed 16 individuals from each cocoon that were maintained socially until five days then kept isolated or in groups. These individuals were not used for behavioral assays. Spiderlings were introduced to 1 ml Eppendorf tube and immersed in liquid nitrogen. The tubes were stored at −80°C until the analysis. Samples were thawed at 20°C for about 10 min. Each sample was then introduced in a 2-ml autosampler vial and washed for five min with 100 μl pentane (Merck, Darmstadt, Germany). After five min, the solution was transferred to a 250-μL autosampler vial for chemical analysis. GC-MS analysis was performed in a mass spectrometer ISQQD Single Quadrupole GC-MS System (Thermo Fisher Scientific Inc., Villebon sur Yvette, France), fitted with a capillary column (Restek RTX-5MS 30 min × 0.25 mm, 0.25 μm film thickness, 5% diphenyl and 95% dimethylpolysiloxane) and a splitless injector (300°C). Ionization was by electron impact (70 eV, source temperature: 250°C). Helium was the carrier gas (1,2 ml/min). After sample injection (2 μl), the oven temperature was maintained at 100°C during two min. then programmed at 10°C/min to 320°C and held for 5 min. The mass spectra were scanned in full scan mode from 50 to 600 m/z values. Samples were automatically injected using an autosampler AS300 (Thermo Fisher Scientific Inc., Villebon sur Yvette, France). For each GC sample, peak heights were calculated by manual integration using the Xcalibur software and were then expressed as the percentage of the total peak height. A total of 55 compounds were considered in the analysis (S5 Table). A standard mixture of alkanes, from n-C7 to n-C40 (Supelco, Sigma-Aldrich, 10 ng/μL each component), was used as a qualitative reference. The identification of the structure of hydrocarbons was determined using the mass spectral fragmentation patterns, comparison of the retention times with those of injected known compounds and the NIST library spectra.

A nonmetric multidimensional scaling (NMDS) based on Bray-Curtis dissimilarities was performed on cuticular hydrocarbons data to visualize the relationships between groups on a two-dimensional layout. The NMDS is an unsupervised technique that ranks the distances to map objects onto a two-dimensional ordination, preserving ranked differences but not the original distances [76]. The quality of the ordination is given by a value of stress ranging between 0 and 1. A stress value of 0.05 reveals an excellent ordination and a value of 0.3 reveals a poor representation. Differences in cuticular lipid profiles between cocoons and treatments were tested using PERMANOVAs (function *adonis* in the R package *vegan*, 999 runs [77]). For each cocoon, we next calculated the Variable Importance in Projection (VIP) scores (1st and 2nd component) of each compound following a discriminant analysis (Partial Least Squares Discriminant Analysis, PLS-DA [78]). High VIP scores indicate highly influential compounds in the discrimination between spiderlings maintained alone or in groups (S9 Fig). The comparison of the compounds with the highest VIP scores between cocoons allowed us to determine whether social isolation induced consistent changes in cuticular profiles.

## Supporting information

S1 Movie Influence of isolation on the nature of social interactions of pairs of 20-day spiderlings. (AVI)

S1 Table Gregariousness is a common trait in solitary spiderlings. This table provides a non-exhaustive list of species of solitary (absence of the mothers when spiderlings emerge from the cocoon) and transient subsocial (maternal care ceases and spiderlings disperse when they start feeding [23]) spiders showing a transient gregarious phase at juvenile stages. The duration of gregariousness is indicated when available. For subsocial species, see [23].
(PDF)

S2 Table Features of the paths of single spiderlings close to walls and far from walls in the dispersal arena. *V* is the average speed, *τ*_*p*_ is characteristic time before an exit, a U-turn or a stop in the external ring, l* is the transport mean free path (i.e., the distance for which the random walk becomes uncorrelated), *τ*_*Stop*,*C*_ is the characteristic time before a stop far from the walls. For stopped spiders, *α* and *β* give the probability of moving (Eq 1). See [5,36,72] for details.
(PDF)

S3 Table Fraction of stopped spiderlings that moved after being collided and fraction of moving spiders that stopped in presence of N individuals. See [5] for details.
(PDF)

S4 Table Pairwise comparisons (chi-square tests with Holm’s correction) of the proportion of cannibalism in siblings of different ages and that experienced different treatments (see Fig 4). D0, D5, D10, D15 or D20 indicates the age at which individuals were tested. “Socially,” “5DSoc_Alone,” and “Alone” indicate that individuals were raised socially, socially until 5 Days then isolated, or in isolation, respectively. “Fed” indicates individuals that were fed three times. Relevant data values are included in S1 Data.
(PDF)

S5 Table List of cuticular compounds in 2nd-instar spiderlings of *A. labyrinthica* raised in social isolation or in groups. Mean and SD of the relative abundance of cuticular compounds. Spiderlings from three cocoons were used (see S8 Fig). “x” indicates uncertain methyl group position. Relevant data values are included in S1 Data. KI, Kovat's indices; Tr: retention time (min).
(PDF)

S1 Fig Influence of the presence of silk on dispersal dynamics. Groups of four spiderlings (*N* = 19) were introduced to dispersal arenas. Four cocoons collected in 2016 (three from Marquefave and one from Canens) were used. Each arena was watered every two days with an atomizer. In nine arenas, we introduced a paintbrush into each hole drilled in the lid of the arena to remove the silk in each quadrant every two days (before watering). Each arena was photographed at 15:00 during 10 consecutive days. We compared the mean nearest-neighbor distances over time between the two groups with an ANOVA-type test using the function *ANOVA*.*test* from the package *nparLD* implemented in R [79]. We found a significant effect of time (*ATS* = 31.13, *df* = 6.01, *P* < 0.001) but no effect of the presence of silk on the dynamics of dispersal (*ATS* = 11.27, *df* = 1, *P* = 0.92), the interaction being also non-significant (*ATS* = 0.73, *df* = 6.01, *P* = 0.62). Relevant data values are included in S1 Data.
(TIF)

S2 Fig Synchronization in the onset of activity. Each point corresponds to the date (time window of 6 h) at which siblings introduced alone or in groups in dispersal arenas were first seen moving substantially. Relevant data values are included in S2 Data.
(TIF)

S3 Fig **Method used to obtain the lifetimes of aggregates and the probability of stopping close to a conspecific in real (red) and virtual (blue) pairs of spiderlings**. The coordinates of paired and isolated spiderlings were obtained using the tracking software SwisTrack (v4.0). After recentering the data, we superimposed the coordinates of two individuals tested alone to create virtual pairs. Relevant data values are included in S1 Data.
(TIF)

S4 Fig Behavioral algorithm implemented in Monte Carlo simulations. The flow chart summarizes all rules and parameters used in simulations to govern the transitions between states. See S2 and S3 Tables for the values of parameters.
(TIF)

S5 Fig **Experimental (blue) and theoretical (orange) patterns of aggregation and mobility**. (A) Fraction (± 95% CI) of time spent moving as a function of day for spiderlings tested alone in experiments (*N* = 14) and simulations (*N* = 1,000). Colored bands are 95% CI. **(**B) Mean interindividual distances on Day 2 in groups of four spiderlings in experiments (*N* = 14) and simulations (*N* = 1,000). Relevant data values are included in S1 and S2 Data.
(TIF)

S6 Fig Experimental design to study the influence of social isolation on aggression and cannibalism. Spiderlings were maintained in isolation or in groups for different durations before they were paired with an unfamiliar sibling. Test arenas were surveyed daily for five consecutive days to record mortality. Video-recordings were used to score the level of aggression for tests at Day 20. A) Paired spiderlings experienced the same social context before being tested. i) Spiderlings were tested at Day 0 or they were maintained in groups until being tested at Day 5, 10, 15, or 20. ii) Spiderlings were tested at Day 0 or raised socially until Day 5. Spiderlings were then either tested at Day 5 or placed alone until being tested at Days 10, 15 or 20. A sample of spiderlings tested a Day 20 were also fed at Days 3, 9, and 15 to estimate the influence of the nutritional state of aggressiveness. iii) Spiderlings were maintained alone and tested at Day 0, 5, 10, 15, or 20. B) Paired spiderlings experienced different social context before being tested at Day 20. In each pair, one spiderling was maintained socially until the test and one spiderling was reared in group for 5 days before being isolated.
(TIF)

S7 Fig Rate of social contacts. Fraction of the time spent at less than 0.6 cm from a conspecific in groups of four spiderlings introduced to 4-compartments arenas (i.e., dispersal arenas). The grey band represents 95% confidence intervals. Relevant data values are included in S2 Data.
(TIF)

S8 Fig **Nonmetric multidimensional scaling (NMDS) plot of the total cuticular extracts of 22-day old spiderlings that have been raised alone (blue) or in groups (yellow)**. Spiderlings from three cocoons collected at two locations (Duras: squares and triangles, Lézat-sur-Lèze: circles) were used. NMDS Stress = 0.08. A non-parametric analysis of variance (Adonis) showed a cocoon effect (F = 129.72, *P* < 0.001) and an interaction between cocoon and treatment (F = 6.14, P = 0.006) but no effect of treatment (F = 0.88, P = 0.375). Relevant data values are included in S1 Data.
(TIF)

S9 Fig Influence of social isolation of cuticular profiles. For each cocoon, a PLS-DA was performed between spiderlings raised alone or in groups to compute the VIP scores of each compound. The symbol above each plot refers to the symbol in S8 Fig. A high score indicates influential compounds in the discrimination between spiderlings that experienced different social contexts. The identity of each compound (C1 to C55) is provided in S5 Table. Relevant data values are included in S1 Data. PLS-DA, Partial Least Squares Discriminant Analysis; VIP, Variable Importance in Projection.
(TIF)

S10 Fig Influence of watering on dispersal dynamics. We verified that the absence of water had no influence on the onset and dynamics of dispersal. Using nine cocoons (five from Ramonville, four from Lézat-sur-Lèze), we introduced groups of four spiderlings (*N* = 30) to dispersal arenas. We watered half of the dispersal arenas every two days with an atomizer. Each arena was photographed at 14:00 during 13 consecutive days. We compared the mean nearest-neighbor distances over time between the two groups with an ANOVA-type test using the function *ANOVA*.*test* from the package *nparLD* implemented in R [79]. We found a significant effect of time (*ATS* = 31.13, *df* = 5.94, *P* < 0.001) but no effect of watering on dispersal dynamics (*ATS* = 0.02, *df* = 1, *P* = 0.87), the interaction being also non-significant (*ATS* = 1.52, *df* = 5.94, *P* = 0.17). Relevant data values are included in S1 Data.
(TIF)

S1 Data Excel file containing the underlying numerical data and statistical analysis for figure panels Figs 1, 3A, 3B, 4A, 4B, 5A, 5B, 6A, 6B and 6C and S1, S2, S8, S9 and S10 Figs. (XLSX)

S2 Data Excel file containing the underlying numerical data and statistical analysis for figure panels Figs 2 and 3B and S2, S5 and S7 Figs. (ZIP)

## Abbreviations

NMDS: nonmetric multidimensional scaling
PLS-DA: Partial Least Squares Discriminant Analysis
VIP: Variable Importance in Projection
