# Supplementary material for: Social intolerance is a consequence, not a cause, of dispersal in spiders
Source: PLoS Biol. 2019 Jul 2;17(7):e3000319. doi: 10.1371/journal.pbio.3000319 (PMC6605646; doi:10.1371/journal.pbio.3000319)
Supplement: S1 Table — This table provides a non-exhaustive list of species of solitary (absence of the mothers when spiderlings emerge from the cocoon) and transient subsocial (maternal care ceases and spiderlings disperse when they start feeding [23]) spiders showing a transient gregarious phase at juvenile stages. The duration of gregariousness is indicated when available. For subsocial species, see [23]. (PDF) [file pbio.3000319.s002.pdf]

S1 Table. Gregariousness is a common trait in solitary spiderlings.

Infraorder	Family	Genus	Species	Level of sociality	Duration	Reference
Mygalomorphae	Antrodiaetidae	*Aliatypus*	*spp.*	Transient subsocial	Probably more than one month	(1)
	Atypidae	*Sphodros*	*fitchi*	Transient subsocial	Unknown	(2)
		Calommata	*signata*	Transient subsocial	2 weeks	(3)
	Ctenizidae	*Stasimopus*	*mandelai*	Transient subsocial	Unknown	(4)
	Cyrtaucheniidae	*Ancyloptrypa*	*sp.*	Transient subsocial	Unknown	(5)
	Migidae	*Clathotarsus*	*simoni*	Transient subsocial	4 months	(6)
	Theraphosidae	*Brachypelma*	*vagans*	Transient subsocial	A few weeks (at least until the 1st molt)	(7, 8)
		Eurypelma	*californicum*	Transient subsocial	1 -2 weeks	(9)
Araneomorphae	Agelenidae	*Agelena*	*labyrinthica*	Solitary	A few days	(10)
	Araneidae	*Araneus*	*diadematus*	Solitary	> 3 days	(11)
		Argiope	*aurantia*	Solitary	3-8 days	(12)
		Argiope	*trifasciata*	Solitary	3-8 days	(12)
		Nephila	*clavipes*	Solitary	Until 3rd molt (and sometime more)	(13)
		Poecilopachys	*australasia*	Solitary	4-13 days	(14)
	Austrochilidae	*Hickmania*	*troglodytes*	Solitary	<1 month	(15)
	Clubionidae	*Clubiona*	*spp.*	Transient subsocial	Unknown	(16)
	Ctenidae	*Cupiennius*	*spp.*	Solitary	1 week	(17)
	Dictynidae	*Dictyna*	*coloradensis*	Solitary	End of 1st instar - beginning of 2nd instar	(18)
	Dysderidae	*Dysdera*	*crocata*	Transient subsocial	Unknown	(19)
	Eresidae	*Stegodyphus*	*africanus*	Solitary / Transient subsocial	> 30 days	(20)
	Hypochilidae	*Hypochilus*	*thorelli*	Solitary	Unknown	(21)
	Lycosidae	*Hogna*	*carolinensis*	Transient subsocial	Until 1 week	(22)
		Lycosa	*georgicola*	Transient subsocial	Unknown	(23)
		Pardosa	*saltans*	Transient subsocial	Around 4 days	(24)
		Schizocosa	*ocreata*	Transient subsocial	1 week	(25)
	Oxyopidae	*Peucetia*	*viridans*	Transient subsocial	A few days	(26, 27)
	Pholcidae	*Crossopriza*	*lyoni*	Transient subsocial	> 2-4 days	(28)
		Holocnemus	*pluchei*	Transient subsocial	Until the 1st molt	(29)

Salticidae	*Portia*	*fimbriata*	Solitary	Unknown	(30)
	Phidippus	*coccineus*	Solitary	2 weeks	(31)
Sicariidae	*Loxosceles*	*gaucho*	Transient subsocial / Subsocial	Unknown	(32)
Tetrablemmidae	*Monoblemma*	*muchmorei*	Transient subsocial	> 1 week	(33)
Theridiidae	*Achaeranea*	*iepidorium*	Solitary	< 1 week	(34)
	Latrodectus	*hasselti*	Transient subsocial	1 week	(35)
	Latrodectus	*hesperus*	Transient subsocial	1 -2 weeks	(36, 37)
Thomisidae	*Misumena*	*vatia*	Solitary / Transient subsocial	Until the 2nd molt	(38)
Zodariidae	*Lutica*	*spp.*	Transient subsocial	Unknown	(39)

This table provides a non-exhaustive list of species of solitary (absence of the mothers when spiderlings emerge from the cocoon) and transient subsocial (maternal care ceases and spiderlings disperse when they start feeding [23]) spiders showing a transient gregarious phase at juvenile stages. The duration of gregariousness is indicated when available. For subsocial species, see [23].

1. Coyle FA, Icenogle WR. Natural history of the Californian trapdoor spider genus *Aliatypus* (Araneae, Antrodiaetidae). J Arachnol. 1994: 225-55.
2. Cutler B, Guarisco H. Dispersal aggregation of *Sphodros fitchi* (Araneae, Atypidae). J Arachnol. 1995;23: 205-6.
3. Kuwada-Kusunose T, Sakai T, Suzuki K. Observations of the early postembryonic development and dispersal of the purse-web spider *Calommata signata* (Araneae: Atypidae). Acta Arachnol. 2016;65: 43-7.
4. Hendrixson BE, Bond JE. A new species of *Stasimopus* from the Eastern Cape province of South Africa (Araneae, Mygalomorphae, Ctenizidae), with notes on its natural history. Zootaxa. 2004;619: 1-14.
5. Leroy A, Leroy J. Notes on the natural history of a trapdoor spider *Ancylotrypa* Simon (Araneae, Cyrtaucheniidae) that constructs a spherical burrow plug. J Arachnol. 2005;33: 558-62.
6. Ferretti N, Copperi S, Schwerdt L, Pompozzi G. Another migid in the wall: natural history of the endemic and rare spider *Calathotarsus simoni* (Mygalomorphae: Migidae) from a hill slope in central Argentina. J Nat Hist. 2014;48: 1907-21.
7. Reichling SB. Group dispersal in juvenile *Brachypelma vagans* (Araneae, Theraphosidae). J Arachnol. 2000;28: 248-51.
8. Dor A, Hénaut Y. Silk use and spiderling behavior in the tarantula *Brachypelma vagans* (Araneae: Theraphosidae). **Acta Zool Mex**. 2012;28.
9. Baerg WJ. Tarantula studies. J New York Entomol S. 1938;46: 31-43.
10. Mougenot F, Combe M, Jeanson R. Ontogenesis and dynamics of aggregation in a solitary spider. Anim Behav. 2012;84: 391-8.
11. Burch TL. The importance of communal experience to survival for spiderlings of *Araneus diadematus* (Araneae: Araneidae). J Arachnol. 1979: 1-18.
12. Tolbert WW. Aerial dispersal behavior of two orb weaving spiders. Psyche. 1977;84: 13-27.
13. Hill EM, Christenson TE. Effects of prey characteristics and web structure on feeding and predatory responses of *Nephila clavipes* spiderlings. Behav Ecol Sociobiol. 1981;8: 1-5.

14. May BM, Gardiner DI. Observations on the Australian "two spined" spider *Poeczlopachys australaszae* in an auckland garden. Weta. 1995;18: 1-5.
15. Doran N, Richardson A, Swain R. The reproductive behaviour of the Tasmanian cave spider *Hickmania troglodytes* (Araneae: Austrochilidae). J Zool. 2001;253: 405-18.
16. Austin A. Life history of *Clubiona robusta* L. Koch and related species (Araneae, Clubionidae) in South Australia. J Arachnol. 1984: 87-104.
17. Schmitt A, Schuster M, Barth FG. Daily locomotor activity patterns in three species of *Cupiennius* (Araneae, Ctenidae): The males are the wandering spiders. J Arachnol. 1990: 249-55.
18. Wheeler G, McCaffrey J, Johnson J. Developmental biology of *Dictyna spp.* (Araneae: Dictynidae) in the laboratory and field. Am Midl Nat. 1990: 124-34.
19. Guarisco H. Three species of house spiders first recorded in Kansas: *Dysdera crocata* (Dysderidae), *Scytodes thoracica* (Scytodidae), and *Cheiracanthium mildei* (Clubionidae). Trans Kans Acad Sci. 1991: 73-6.
20. Seibt U, Wickler I, Wickler W. Dispersal in the solitary Stegodyphus africanus and heterospecific grouping with the social *Stegodyphus dumicola* (Araneae, Eresidae). J Arachnol. 1998: 97-100.
21. Fergusson IC. Natural history of the spider *Hypochilus thorelli* Marx (Hypochilidae). Psyche. 1972;79: 179-99.
22. Punzo F, Alvarez J. Effects of early contact with maternal parent on locomotor activity and exploratory behavior in spiderlings of *Hogna carolinensis* (Araneae: Lycosidae). J Insect Behav. 2002;15: 455-65.
23. Miller GL. Subsocial organization and behavior in broods of the obligate burrowing wolf spider *Geolycosa turricola* (Treat). Can J Zool. 1989;67: 819-24.
24. Ruhland F, Pétillon J, Trabalon M. Physiological costs during the first maternal care in the wolf spider *Pardosa saltans* (Araneae, Lycosidae). J Insect Physiol. 2016;95: 42-50.
25. Toft S, Wise DH. Behavioral and ecophysiological responses of a generalist predator to single-and mixed-species diets of different quality. Oecologia. 1999;119: 198-207.
26. Willey MB, Adler PH. Biology of *Peucetia viridans* (Araneae, Oxyopidae) in South Carolina, with special reference to predation and maternal care. J Arachnol. 1989: 275-84.
27. Fink LS. Costs and benefits of maternal behaviour in the green lynx spider (Oxyopidae, *Peucetia viridans*). Anim Behav. 1986;34: 1051-60.
28. Strickman D, Sithiprasasna R, Southard D. Bionomics of the spider, *Crossopriza lyoni* (Araneae, Pholcidae), a predator of dengue vectors in Thailand. J Arachnol. 1997: 194-201.
29. Jakob EM, Dingle H. Food level and life history characteristics in a pholcid spider (*Holocnemus pluchei*). Psyche. 1990;97: 95-110.
30. Jackson R, Blest A. The biology of *Portia fimbriata*, a web-building jumping spider (Araneae, Salticidae) from Queensland: utilization of webs and predatory versatility. J Zool. 1982;196: 255-93.
31. Gardner BT. Observations on three species of *Phidippus* jumping spiders (Araneae: Salticidae). Psyche. 1965;72: 133-47.

32. Japyassú HF, Macagnan CR, Knysak I. Eggsac recognition in *Loxosceles gaucho* (Araneae, Sicariidae) and the evolution of maternal care in spiders. J Arachnol. 2003;31: 90-105.
33. Edwards RL, Edwards AD. Life history and ecology of the armored spider *Monoblemma muchmorei* (Araneae, Tetrablemmidae). J Arachnol. 2006;34: 599-610.
34. Valerio CE. A unique case of mutualism. Am Nat. 1975;109: 235-8.
35. Modanu M, Li LDX, Said H, Rathitharan N, Andrade MC. Sibling cannibalism in a web-building spider: Effects of density and shared environment. Behav Process. 2014;106: 12-6.
36. Salomon M, Vibert S, Bennett RG. Habitat use by western black widow spiders (*Latrodectus hesperus*) in coastal British Columbia: evidence of facultative group living. Can J Zool. 2010;88: 334-46.
37. Johnson JC, Kitchen K, Andrade MC. Family affects sibling cannibalism in the black widow spider, *Latrodectus hesperus*. Ethology. 2010;116: 770-7.
38. Morse DH. Do cannibalism and kin recognition occur in just-emerged crab spiderlings? J Arachnol. 2011;39: 53-9.
39. Ramirez MG. Natural history of the spider genus *Lutica* (Araneae, Zodariidae). J Arachnol. 1995;23: 111-7.
