# Supplementary material for: Social intolerance is a consequence, not a cause, of dispersal in spiders
Source: PLoS Biol. 2019 Jul 2;17(7):e3000319. doi: 10.1371/journal.pbio.3000319 (PMC6605646; doi:10.1371/journal.pbio.3000319)
Supplement: S2 Table — V is the average speed, τp is characteristic time before an exit, a U-turn or a stop in the external ring, l* is the transport mean free path (i.e., the distance for which the random walk becomes uncorrelated), τStop,C is the characteristic time before a stop far from the walls. For stopped spiders, α and β give the probability of moving (Eq 1). See [5,36,72] for details. (PDF) [file pbio.3000319.s003.pdf]

S2 Table. Features of the paths of single spiderlings close to walls and far from walls in the dispersal arena.

	Moving state										
	Close to walls					Far from walls				Stopped state	
Days	V_P (mm/s)	$1/\tau_P$ (1/s)	% Exit	% U-turn	% Stop	V_C (mm/s)	l^* (mm)	$1/\tau_{Stop,C}$ (1/s)	Days	α	β
0 to 11	6.5	0.196	10	61	29	6.5	10.66	0.023	0 to 3	0.48	0.74
									4 to 11	0.936	0.74

V is the average speed, τ_p is characteristic time before an exit, a U-turn or a stop in the external ring, l^* is the transport mean free path (i.e., the distance for which the random walk becomes uncorrelated), $\tau_{Stop,C}$ is the characteristic time before a stop far from the walls. For stopped spiders, α and β give the probability of moving (Eq 1). See [5,36,72] for details.
