# Supplementary material for: Social intolerance is a consequence, not a cause, of dispersal in spiders
Source: PLoS Biol. 2019 Jul 2;17(7):e3000319. doi: 10.1371/journal.pbio.3000319 (PMC6605646; doi:10.1371/journal.pbio.3000319)
Supplement: S3 Table — See [5] for details. (PDF) [file pbio.3000319.s004.pdf]

S3 Table. Fraction of stopped spiderlings that moved after being collided and fraction of moving spiders that stopped in presence of N individuals. See [5] for details.

Size of the aggregate	$F_{Collision}$	F_{Stop}
N=1	0.34	0.27
N=2	0.19	0.37
N=3	0.07	0.55
