# Supplementary material for: Social intolerance is a consequence, not a cause, of dispersal in spiders
Source: PLoS Biol. 2019 Jul 2;17(7):e3000319. doi: 10.1371/journal.pbio.3000319 (PMC6605646; doi:10.1371/journal.pbio.3000319)
Supplement: S4 Table — D0, D5, D10, D15 or D20 indicates the age at which individuals were tested. “Socially,” “5DSoc_Alone,” and “Alone” indicate that individuals were raised socially, socially until 5 Days then isolated, or in isolation, respectively. “Fed” indicates individuals that were fed three times. Relevant data values are included in S1 Data. (PDF) [file pbio.3000319.s005.pdf]

S4 Table. Pairwise comparisons (chi-square tests with Holm's correction) of the proportion of cannibalism in siblings of different ages and that experienced different treatments (see Fig 4).

	D0_Alone	D0_5DSoc_Alone	D0_Socially	D5_Alone	D5_5DSoc_Alone	D5_Socially	D10_Alone	D10_5DSoc_Alone	D10_Socially	D15_Alone	D15_5DSoc_Alone	D15_Socially	D20_Alone	D20_5DSoc_Alone	D20_Socially
D20_5DSoc_Alone_Fed	1.000	1.000	**0.028**	**0.011**	**0.007**	**0.001**	1.000	0.230	**0.009**	1.000	1.000	0.107	1.000	1.000	1.000
D20_Socially	1.000	1.000	1.000	1.000	1.000	1.000	1.000	1.000	1.000	**0.039**	1.000	1.000	**0.000**	**0.010**	
D20_5DSoc_Alone	**0.001**	**0.000**	**0.002**	**0.001**	**0.001**	**0.000**	0.281	**0.024**	0.001	1.000	1.000	**0.010**	1.000		
D20_Alone	**0.000**	**0.000**	**0.000**	**0.000**	**0.000**	**0.000**	**0.008**	**0.000**	**0.000**	1.000	1.000	**0.000**			
D15_Socially	1.000	1.000	1.000	1.000	1.000	1.000	1.000	1.000	1.000	**0.039**	0.379				
D15_5DSoc_Alone	1.000	1.000	0.106	**0.047**	**0.030**	**0.007**	1.000	0.779	**0.038**	1.000					
D15_Alone	**0.003**	**0.001**	**0.009**	**0.004**	**0.003**	**0.001**	0.824	0.088	**0.004**						
D10_Socially	1.000	1.000	1.000	1.000	1.000	1.000	1.000	1.000							
D10_5DSoc_Alone	1.000	1.000	1.000	1.000	1.000	1.000	1.000								
D10_Alone	1.000	1.000	1.000	1.000	1.000	1.000									
D5_Socially	1.000	1.000	1.000	1.000	1.000										
D5_5DSoc_Alone	1.000	1.000	1.000	1.000											
D5_Alone	1.000	1.000	1.000												
D0_Socially	1.000	1.000													
D0_5DSoc_Alone	1.000														

D0, D5, D10, D15 or D20 indicates the age at which individuals were tested. "Socially," "5DSoc_Alone," and "Alone" indicate that individuals were raised socially, socially until 5 Days then isolated, or in isolation, respectively. "Fed" indicates individuals that were fed three times. Relevant data values are included in S1 Data.
