# Supplementary material for: Social intolerance is a consequence, not a cause, of dispersal in spiders
Source: PLoS Biol. 2019 Jul 2;17(7):e3000319. doi: 10.1371/journal.pbio.3000319 (PMC6605646; doi:10.1371/journal.pbio.3000319)
Supplement: S5 Table — Mean and SD of the relative abundance of cuticular compounds. Spiderlings from three cocoons were used (see S8 Fig). “x” indicates uncertain methyl group position. Relevant data values are included in S1 Data. KI, Kovat's indices; Tr: retention time (min). (PDF) [file pbio.3000319.s006.pdf]

**S5 Table. List of cuticular compounds in 2nd-instar spiderlings of A. labyrinthica raised in social isolation or in groups.**

| No. Pic | Identification | Isolated | | Grouped | | Tr | KI |
|---|---|---|---|---|---|---|---|
| | | Mean | SD | Mean | SD | | |
| 1 | nC23 | 0.13 | 0.068 | 0.165 | 0.121 | 17.48 | 2300 |
| 2 | 2,x-dimethylC23 | 0.145 | 0.055 | 0.19 | 0.116 | 18.3 | 2412 |
| 3 | 2,y-dimethylC23 | 0.157 | 0.053 | 0.236 | 0.151 | 18.35 | 2417 |
| 4 | 2-MethylC24 | 0.02 | 0.028 | 0.024 | 0.04 | 18.8 | 2468 |
| 5 | nC25 | 3.86 | 1.214 | 3.768 | 1.087 | 19.1 | 2500 |
| 6 | 11,12-diMethylC24 | 0.204 | 0.094 | 0.209 | 0.095 | 19.36 | 2535 |
| 7 | 7-MethylC25 | 0.079 | 0.036 | 0.072 | 0.046 | 19.42 | 2542 |
| 8 | 5-MethylC25 | 0.061 | 0.025 | 0.068 | 0.057 | 19.49 | 2552 |
| 9 | 2-MethylC25 | 0.08 | 0.08 | 0.076 | 0.078 | 19.58 | 2564 |
| 10 | 3-MethylC25 | 0.226 | 0.039 | 0.233 | 0.09 | 19.66 | 2574 |
| 11 | 9,x-dimethylC25 | 0.2 | 0.084 | 0.198 | 0.074 | 19.72 | 2581 |
| 12 | nC26 | 1.249 | 0.19 | 1.3 | 0.292 | 19.86 | 2600 |
| 13 | 2,y-dimethylC25 | 0.08 | 0.03 | 0.083 | 0.049 | 19.98 | 2616 |
| 14 | 11,x-dimethylC25 | 0.055 | 0.04 | 0.045 | 0.041 | 20.08 | 2629 |
| 15 | 11,12-diMethylC25 | 0.177 | 0.039 | 0.18 | 0.03 | 20.13 | 2636 |
| 16 | 7-MethylC26 | 0.021 | 0.025 | 0.029 | 0.035 | 20.2 | 2646 |
| 17 | 2-MethylC26 | 0.537 | 0.22 | 0.592 | 0.352 | 20.33 | 2663 |
| 18 | 3-MethylC26 | 0.078 | 0.034 | 0.085 | 0.04 | 20.41 | 2674 |
| 19 | nC27 | 21.809 | 2.676 | 21.581 | 2.493 | 20.6 | 2700 |
| 20 | 11,12-diMethylC26 | 9.46 | 3.86 | 9.406 | 2.645 | 20.84 | 2733 |
| 21 | 7-MethylC27 | 3.773 | 1.779 | 3.711 | 1.055 | 20.9 | 2742 |
| 22 | 5-MethylC27 | 1.295 | 0.414 | 1.392 | 0.416 | 20.97 | 2751 |
| 23 | 2-MethylC27 | 2.027 | 2.569 | 2.572 | 3.227 | 21.05 | 2763 |
| 24 | 3-MethylC27 | 14.05 | 4.783 | 13.55 | 3.832 | 21.13 | 2774 |
| 25 | nC28 | 1.21 | 0.295 | 1.242 | 0.316 | 21.32 | 2800 |
| 26 | 8,x-dimethylC27 | 0.466 | 0.176 | 0.536 | 0.177 | 21.36 | 2805 |
| 27 | 8,y-dimethylC27 | 0.37 | 0.144 | 0.363 | 0.179 | 21.38 | 2808 |
| 28 | 11,12-diMethylC27 | 0.765 | 0.295 | 0.807 | 0.285 | 21.55 | 2833 |
| 29 | 7-MethylC28 x-MethylC28:1 | 0.555 | 0.894 | 0.279 | 0.294 | 21.59 | 2840 |
| 30 | 2-MethylC28 | 2.304 | 1.303 | 2.298 | 1.212 | 21.75 | 2863 |
| 31 | 3-MethylC28 | 0.281 | 0.113 | 0.301 | 0.117 | 21.82 | 2872 |
| 32 | nC29 | 5.191 | 2.278 | 4.938 | 0.996 | 22.01 | 2900 |
| 33 | 11,12-diMethylC28 | 6.606 | 1.755 | 7.265 | 2.397 | 22.23 | 2933 |
| 34 | 7-MethylC29 | 2.683 | 0.981 | 2.584 | 0.652 | 22.28 | 2941 |
| 35 | 5-MethylC29 | 1.21 | 0.159 | 1.215 | 0.148 | 22.35 | 2951 |
| 36 | 11,y-dimethylC29 | 1.728 | 0.691 | 1.616 | 0.843 | 22.4 | 2959 |
| 37 | 2-MethylC29 | 2.48 | 1.093 | 2.435 | 1.025 | 22.43 | 2963 |
| 38 | 11,x-dimethylC29 | 0.2 | 0.436 | 0.418 | 0.659 | 22.47 | 2969 |
| 39 | 3-MethylC29 | 4.194 | 1.926 | 3.906 | 1.613 | 22.51 | 2975 |
| 40 | NI | 0.093 | 0.169 | 0.122 | 0.129 | 22.63 | 2994 |
| 41 | nC30 | 0.382 | 0.33 | 0.349 | 0.159 | 22.68 | 3000 |
| 42 | 8,x-dimethylC29 | 0.459 | 0.103 | 0.458 | 0.061 | 22.72 | 3008 |
| 43 | 11,12-diMethylC29 | 0.412 | 0.101 | 0.454 | 0.124 | 22.89 | 3033 |
| 44 | 2-MethylC30 | 0.768 | 0.302 | 0.836 | 0.228 | 23.08 | 3063 |
| 45 | nC31 | 0.495 | 0.353 | 0.416 | 0.14 | 23.32 | 3100 |
| 46 | 11,12-diMethylC30 | 1.623 | 1.014 | 1.68 | 0.635 | 23.52 | 3132 |
| 47 | 7-MethylC31 | 0.505 | 0.186 | 0.532 | 0.311 | 23.58 | 3140 |
| 48 | 11,y-dimethylC31 | 2.114 | 1.129 | 2.121 | 0.731 | 23.68 | 3157 |
| 49 | 9,x-dimethylC25 | 0.419 | 0.118 | 0.408 | 0.108 | 23.83 | 3181 |
| 50 | nC32 | 0.095 | 0.059 | 0.115 | 0.041 | 23.99 | 3200 |
| 51 | 11,x-dimethylC31 | 0.174 | 0.149 | 0.183 | 0.112 | 24.14 | 3229 |
| 52 | 5-MethylC32 | 0.655 | 0.338 | 0.738 | 0.244 | 24.3 | 3255 |
| 53 | 11,x-dimethylC32 | 0.355 | 0.28 | 0.344 | 0.146 | 24.79 | 3328 |
| 54 | 5-MethylC33 | 0.774 | 0.706 | 0.625 | 0.366 | 24.97 | 3354 |
| 55 | 11,x-dimethylC33 | 0.67 | 0.461 | 0.652 | 0.391 | 25.7 | 3424 |

Mean and SD of the relative abundance of cuticular compounds. Spiderlings from three cocoons were used (see S8 Fig). “x” indicates uncertain methyl group position. Relevant data values are included in S1 Data. KI, Kovat's indices; Tr: retention time (min).
