# Supplementary figures and images for: Social intolerance is a consequence, not a cause, of dispersal in spiders
Source: PLoS Biol. 2019 Jul 2;17(7):e3000319. doi: 10.1371/journal.pbio.3000319 (PMC6605646; doi:10.1371/journal.pbio.3000319)

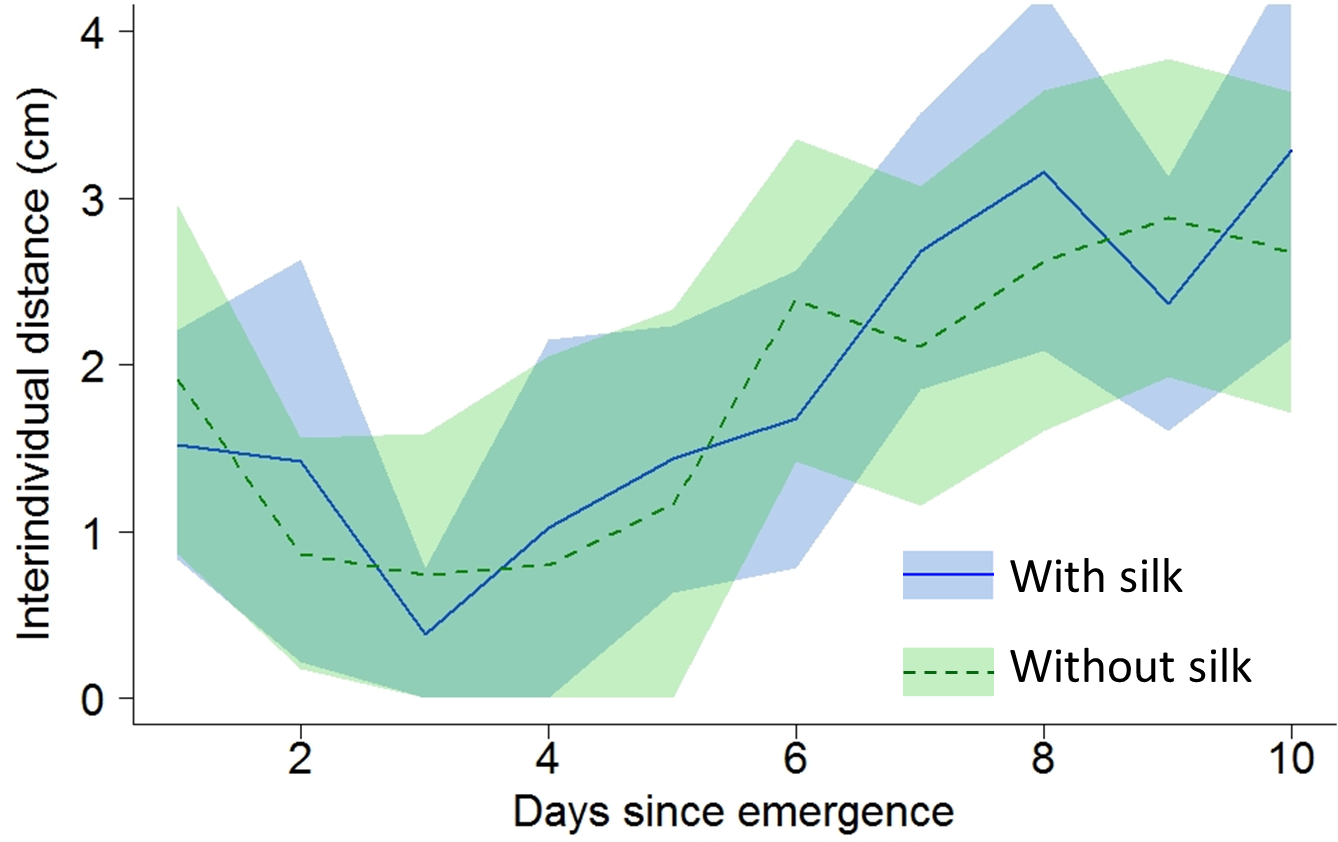

Supplement: S1 Fig — Groups of four spiderlings (N = 19) were introduced to dispersal arenas. Four cocoons collected in 2016 (three from Marquefave and one from Canens) were used. Each arena was watered every two days with an atomizer. In nine arenas, we introduced a paintbrush into each hole drilled in the lid of the arena to remove the silk in each quadrant every two days (before watering). Each arena was photographed at 15:00 during 10 consecutive days. We compared the mean nearest-neighbor distances over time between the two groups with an ANOVA-type test using the function ANOVA.test from the package nparLD implemented in R [79]. We found a significant effect of time (ATS = 31.13, df = 6.01, P < 0.001) but no effect of the presence of silk on the dynamics of dispersal (ATS = 11.27, df = 1, P = 0.92), the interaction being also non-significant (ATS = 0.73, df = 6.01, P = 0.62). Relevant data values are included in S1 Data. (TIF) [file pbio.3000319.s007.tif]

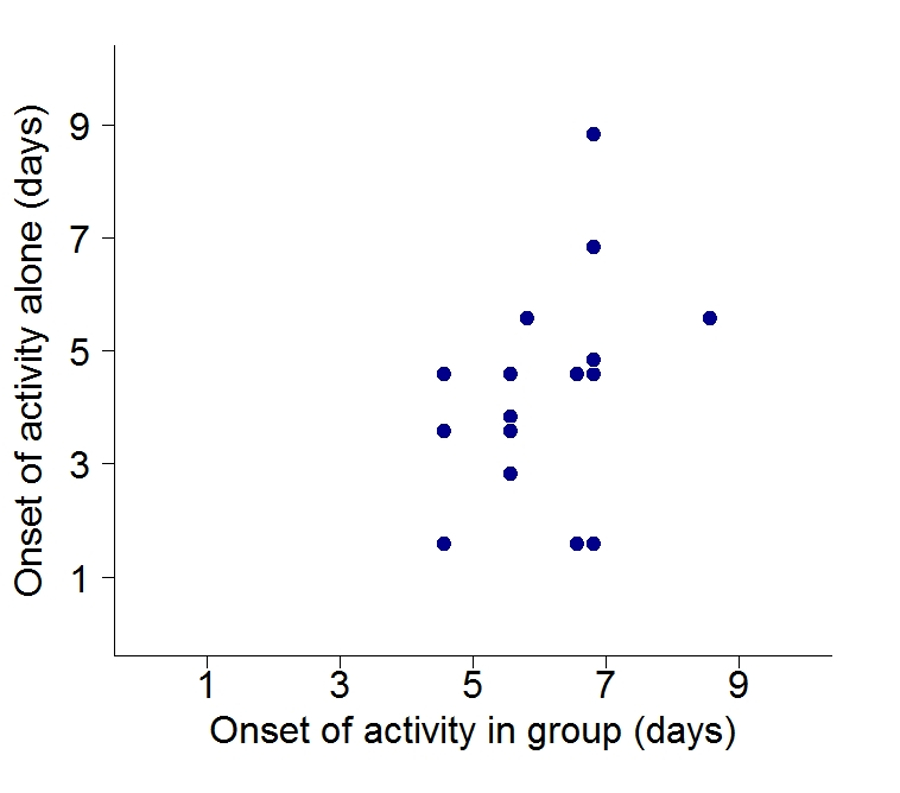

Supplement: S2 Fig — Each point corresponds to the date (time window of 6 h) at which siblings introduced alone or in groups in dispersal arenas were first seen moving substantially. Relevant data values are included in S2 Data. (TIF) [file pbio.3000319.s008.tif]

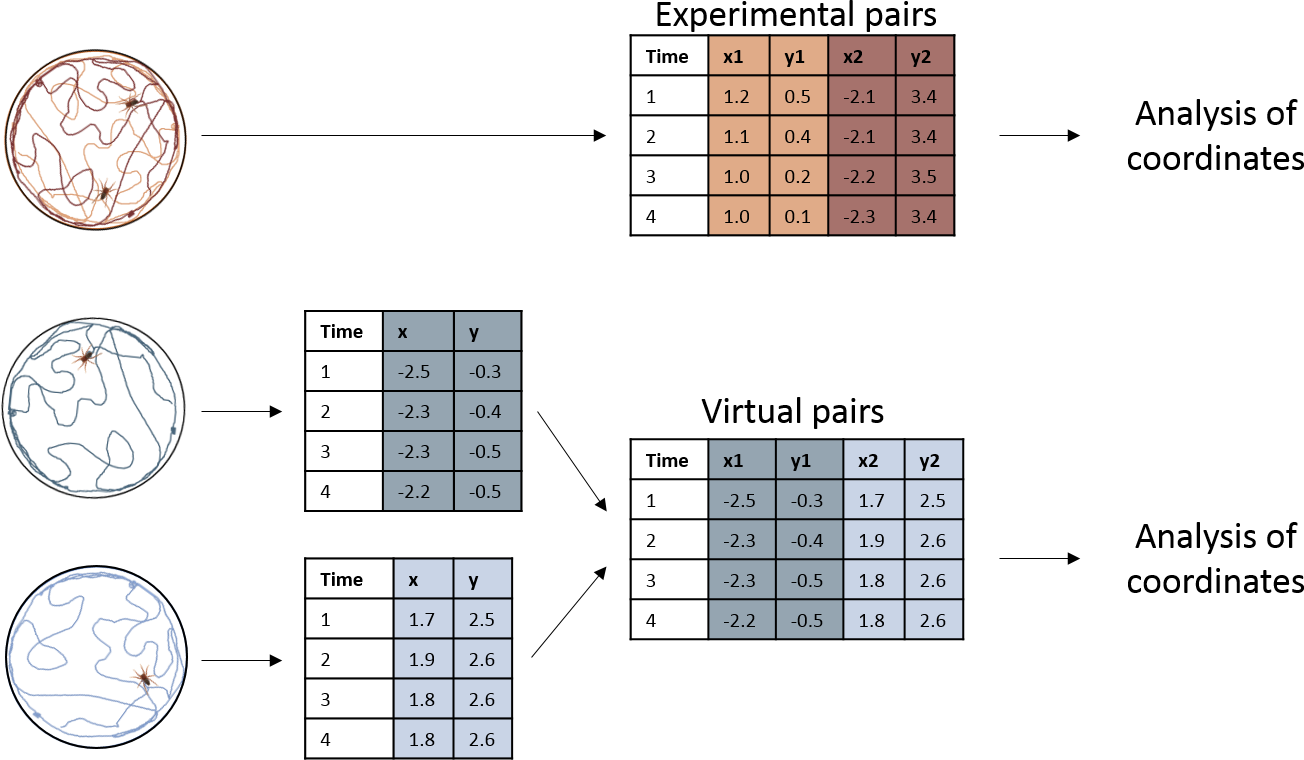

Supplement: S3 Fig — Method used to obtain the lifetimes of aggregates and the probability of stopping close to a conspecific in real (red) and virtual (blue) pairs of spiderlings. The coordinates of paired and isolated spiderlings were obtained using the tracking software SwisTrack (v4.0). After recentering the data, we superimposed the coordinates of two individuals tested alone to create virtual pairs. Relevant data values are included in S1 Data. (TIF) [file pbio.3000319.s009.tif]

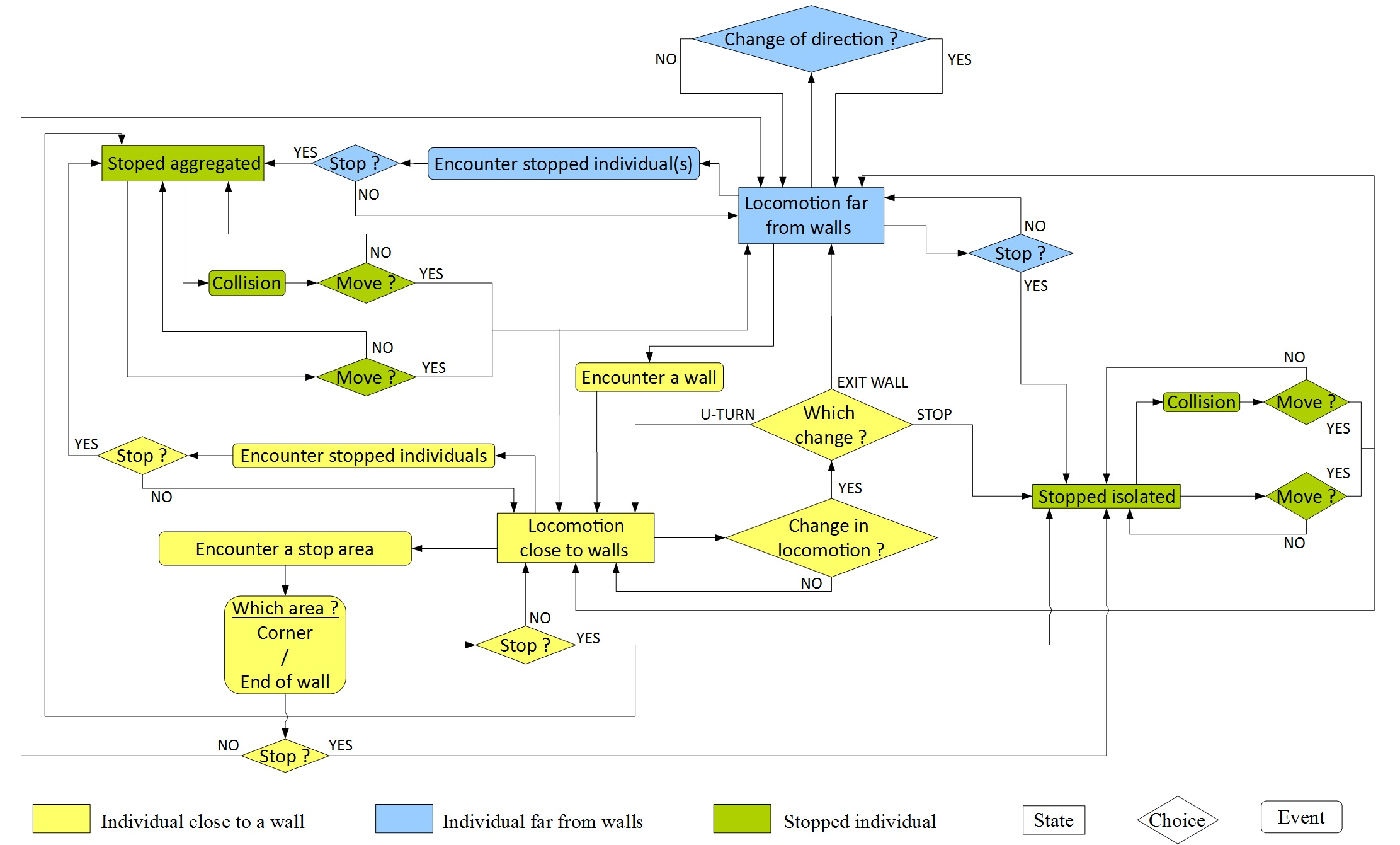

Supplement: S4 Fig — The flow chart summarizes all rules and parameters used in simulations to govern the transitions between states. See S2 and S3 Tables for the values of parameters. (TIF) [file pbio.3000319.s010.tif]

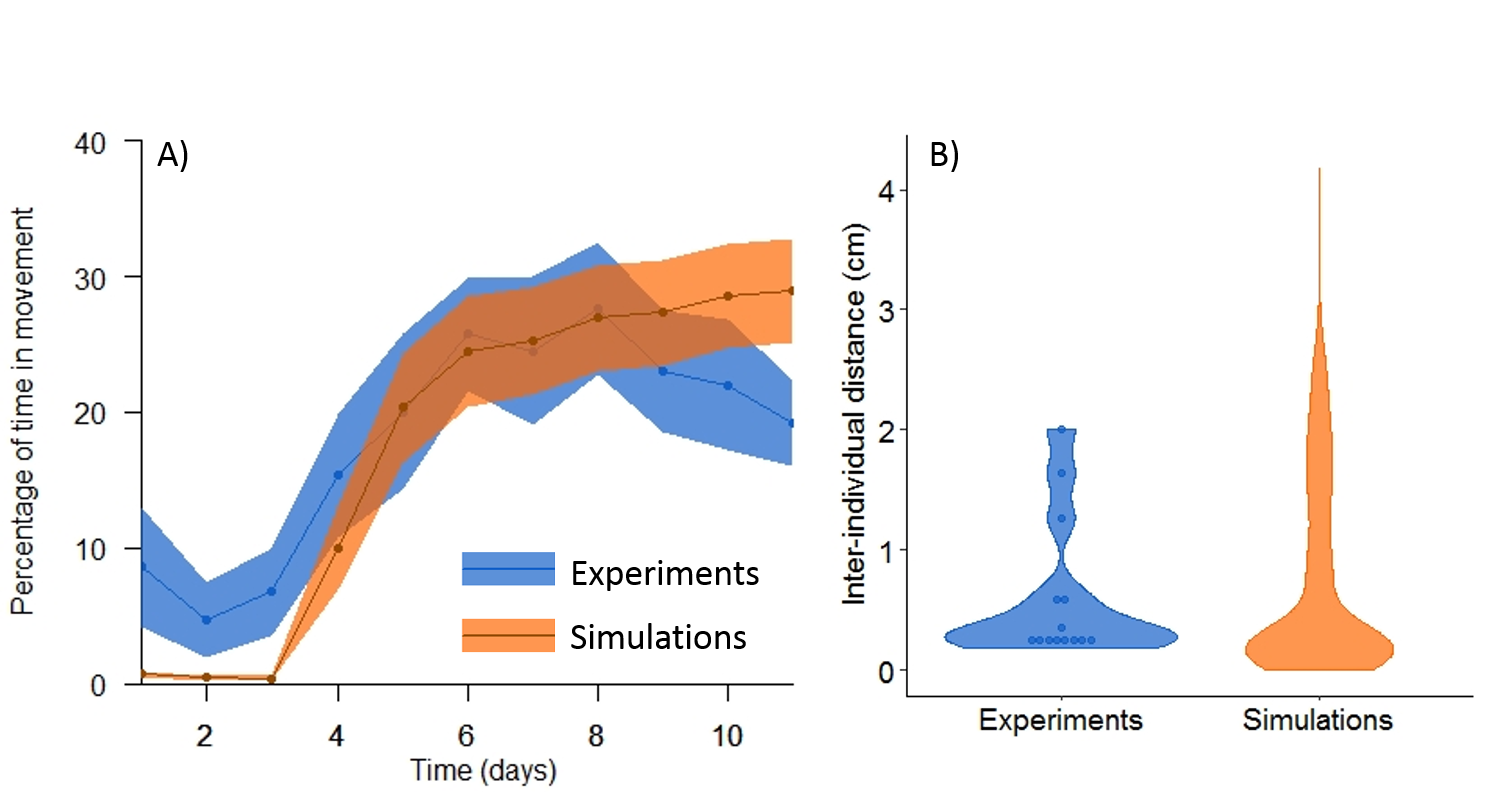

Supplement: S5 Fig — Experimental (blue) and theoretical (orange) patterns of aggregation and mobility. (A) Fraction (± 95% CI) of time spent moving as a function of day for spiderlings tested alone in experiments (N = 14) and simulations (N = 1,000). Colored bands are 95% CI. (B) Mean interindividual distances on Day 2 in groups of four spiderlings in experiments (N = 14) and simulations (N = 1,000). Relevant data values are included in S1 and S2 Data. (TIF) [file pbio.3000319.s011.tif]

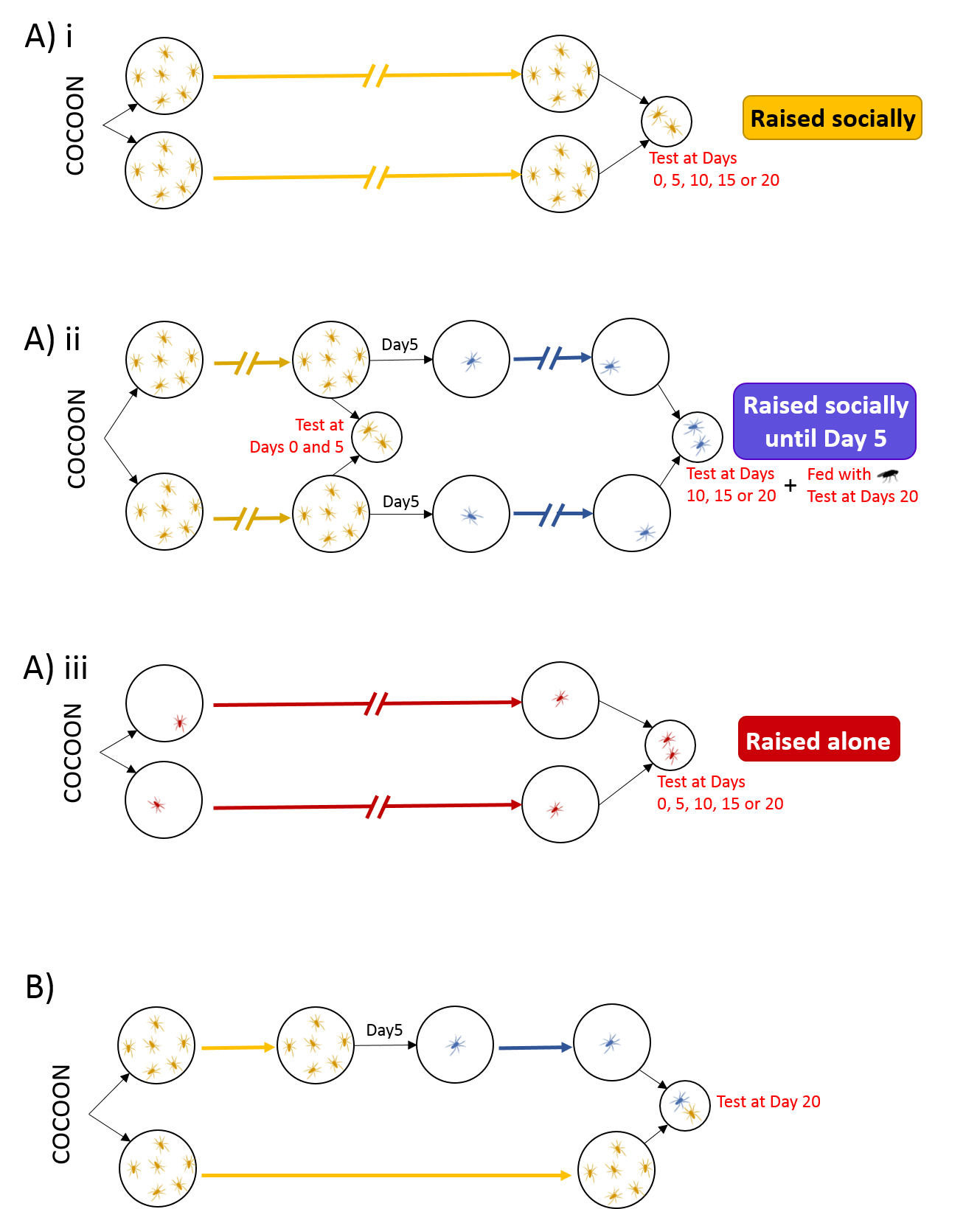

Supplement: S6 Fig — Spiderlings were maintained in isolation or in groups for different durations before they were paired with an unfamiliar sibling. Test arenas were surveyed daily for five consecutive days to record mortality. Video-recordings were used to score the level of aggression for tests at Day 20. A) Paired spiderlings experienced the same social context before being tested. i) Spiderlings were tested at Day 0 or they were maintained in groups until being tested at Day 5, 10, 15, or 20. ii) Spiderlings were tested at Day 0 or raised socially until Day 5. Spiderlings were then either tested at Day 5 or placed alone until being tested at Days 10, 15 or 20. A sample of spiderlings tested a Day 20 were also fed at Days 3, 9, and 15 to estimate the influence of the nutritional state of aggressiveness. iii) Spiderlings were maintained alone and tested at Day 0, 5, 10, 15, or 20. B) Paired spiderlings experienced different social context before being tested at Day 20. In each pair, one spiderling was maintained socially until the test and one spiderling was reared in group for 5 days before being isolated. (TIF) [file pbio.3000319.s012.tif]

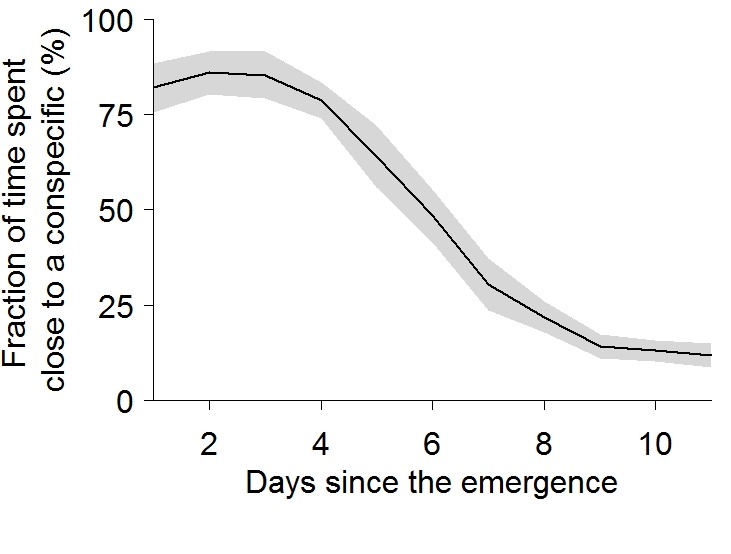

Supplement: S7 Fig — Fraction of the time spent at less than 0.6 cm from a conspecific in groups of four spiderlings introduced to 4-compartments arenas (i.e., dispersal arenas). The grey band represents 95% confidence intervals. Relevant data values are included in S2 Data. (TIF) [file pbio.3000319.s013.tif]

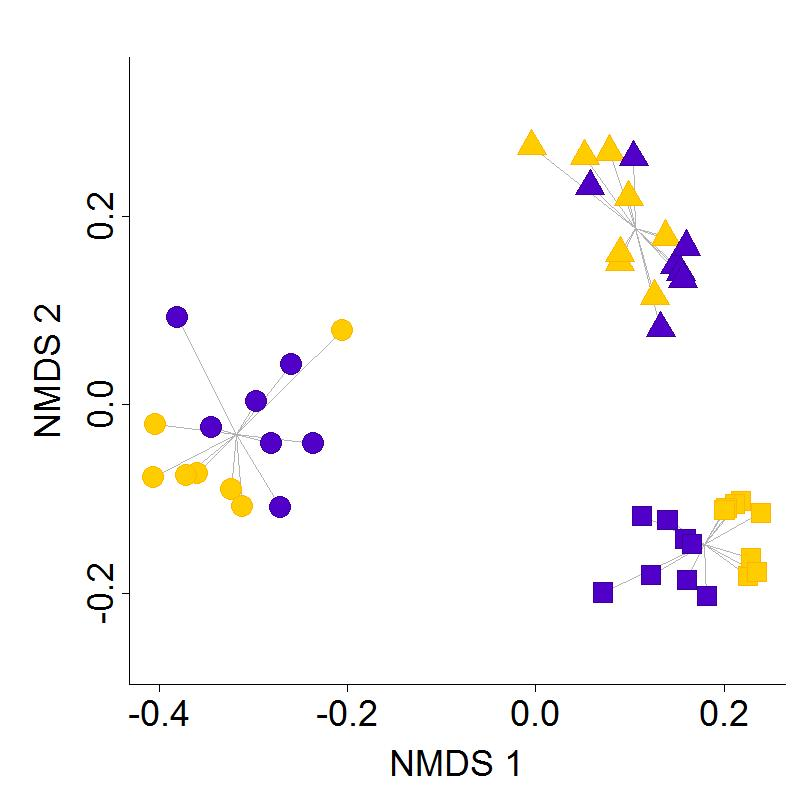

Supplement: S8 Fig — Nonmetric multidimensional scaling (NMDS) plot of the total cuticular extracts of 22-day old spiderlings that have been raised alone (blue) or in groups (yellow). Spiderlings from three cocoons collected at two locations (Duras: squares and triangles, Lézat-sur-Lèze: circles) were used. NMDS Stress = 0.08. A non-parametric analysis of variance (Adonis) showed a cocoon effect (F = 129.72, P < 0.001) and an interaction between cocoon and treatment (F = 6.14, P = 0.006) but no effect of treatment (F = 0.88, P = 0.375). Relevant data values are included in S1 Data. (TIF) [file pbio.3000319.s014.tif]

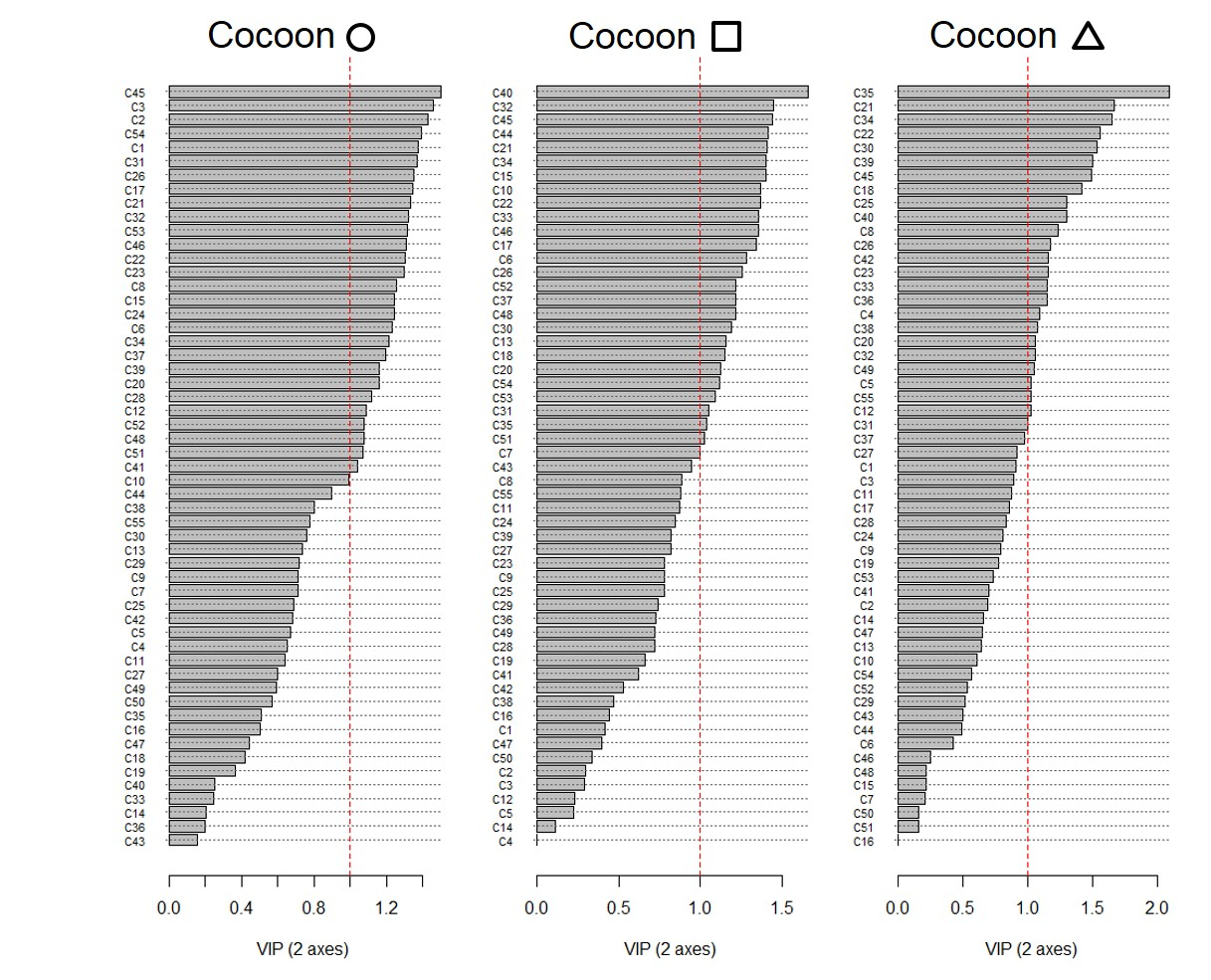

Supplement: S9 Fig — For each cocoon, a PLS-DA was performed between spiderlings raised alone or in groups to compute the VIP scores of each compound. The symbol above each plot refers to the symbol in S8 Fig. A high score indicates influential compounds in the discrimination between spiderlings that experienced different social contexts. The identity of each compound (C1 to C55) is provided in S5 Table. Relevant data values are included in S1 Data. PLS-DA, Partial Least Squares Discriminant Analysis; VIP, Variable Importance in Projection. (TIF) [file pbio.3000319.s015.tif]

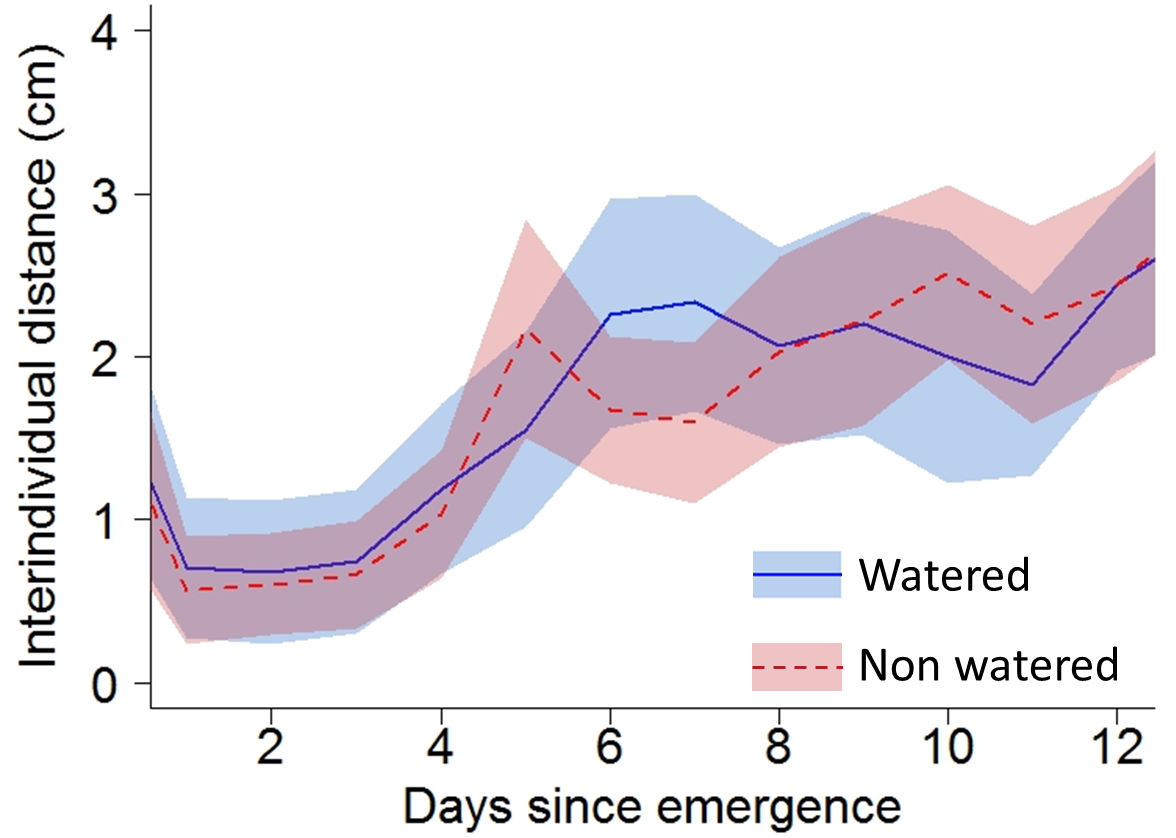

Supplement: S10 Fig — We verified that the absence of water had no influence on the onset and dynamics of dispersal. Using nine cocoons (five from Ramonville, four from Lézat-sur-Lèze), we introduced groups of four spiderlings (N = 30) to dispersal arenas. We watered half of the dispersal arenas every two days with an atomizer. Each arena was photographed at 14:00 during 13 consecutive days. We compared the mean nearest-neighbor distances over time between the two groups with an ANOVA-type test using the function ANOVA.test from the package nparLD implemented in R [79]. We found a significant effect of time (ATS = 31.13, df = 5.94, P < 0.001) but no effect of watering on dispersal dynamics (ATS = 0.02, df = 1, P = 0.87), the interaction being also non-significant (ATS = 1.52, df = 5.94, P = 0.17). Relevant data values are included in S1 Data. (TIF) [file pbio.3000319.s016.tif]
